# Supplementary material for: Dynamic pathogen detection and social feedback shape collective hygiene in ants
Source: Nat Commun. 2023 Jun 3;14:3232. doi: 10.1038/s41467-023-38947-y (PMC10239465; doi:10.1038/s41467-023-38947-y)
Supplement: Supplementary file 1 — Supplementary Information [file 41467_2023_38947_MOESM1_ESM.pdf]

Supplementary information for
Dynamic pathogen detection and social feedback shape collective hygiene in ants

Barbara Casillas-Pérez, Katarína Boďová, Anna V. Grasse, Gašper Tkačik, Sylvia Cremer

*Correspondence to: gasper.tkacik@ist.ac.at, sylvia.cremer@ist.ac.at.

Contents

Supplementary Table 1 **2**
Supplementary Table 2 **3**
Supplementary Table 3 **4**
Supplementary Table 4 **5**
Supplementary Figure 1 **6**
Supplementary Figure 2 **7**
Supplementary Figure 3 **8**
Supplementary Figure 4 **9**
Supplementary Figure 5 **10**
Supplementary Figure 6 **11**
Supplementary Figure 7 **12**
Supplementary Figure 8 **13**
Supplementary Figure 9 **14**
Supplementary Figure 10 **15**
Supplementary Figure 11 **16**
Supplementary Note 1: Model of collective spore removal activity of ants **17**
Supplementary Note 2: Stochastic simulations results **29**
Supplementary Note 3: Statistical data analysis **42**
Supplementary References **53**

Supplementary Table 1

Group treatment		Individual treatment					
		F		f		C	N
		GFP	RFP	GFP	RFP		
FF	17	17	17				68
Ff	16	9	7	7	9		64
ff	16			16	16		64
FC	16	8	8			16	68
fC	17			1	16	17	68
CC	17					34	68
TOTAL	99	66		65		67	396

Supplementary Table 1: **Experimental groups.** Replicate numbers for individual ants and treatment groups. For groups containing two pathogen-treated individuals, we randomized the fluorescent label (GFP, RFP). Together, 198 ants were treated with either the high F or the low f dose of *M. robertsii* or the control solution (C). Together with the 396 nestmates, this leads to a total of 594 analyzed ants.

Supplementary Table 2

behavior		F	f	C	N
selfgrooming of body	V	205	358	469	917
	p	0.00005	0.023	0.105	$\leq 1e^{-11}$
	rc	0.665	0.391	0.264	0.629
selfgrooming of head	V	252	428	444	541
	p	0.0003	0.067	0.078	$\leq 1e^{-11}$
	rc	0.589	0.301	0.304	0.781
poison uptake behavior	V	95	303	359	4700
	p	$\leq 1e^{-11}$	0.006	0.020	$\leq 1e^{-11}$
	rc	0.838	0.485	0.414	-0.899
allogrooming performed	V	899	940	966	106
	p	0.00003	0.0002	0.0003	$\leq 1e^{-11}$
	rc	-0.737	-0.667	-0.643	0.957
allogrooming received	V	12	1	55	4715
	p	$\leq 1e^{-11}$	$\leq 1e^{-11}$	$\leq 1e^{-11}$	$\leq 1e^{-11}$
	rc	0.980	0.998	0.914	-0.905

Supplementary Table 2: **Statistical reporting for ant behavior.** Behavioral changes of ants from the pre- to post-treatment period for individual hygiene, i.e. selfgrooming the body (where treatment was applied; Fig. 1b), and head (which can get contaminated during treatment, self- or allogrooming), as well as poison uptake behavior (which allows usage of formic acid-rich poison in disinfection; Suppl. Fig. 2), and social sanitary care, i.e. performed (Fig. 1c) and received (Fig. 1d) allogrooming. We compare the behavioral changes in the ants according to individual treatment with the high (F) or low (f) pathogen load, the control treatment (C), and for untreated nestmates (N) by paired Wilcoxon tests. The table contains the test statistic V, the two-sided p-value, adjusted for multiple testing, and the effect size (matched-pairs rank biserial correlation coefficient rc, with a positive sign indicating increased performance of the behavior after treatment, and negative sign a decrease); degrees of freedom: $df = 1$ for all tests.

Supplementary Table 3

$v = 5.22, k = 50119$	**Experimental measurement**	**Type II FRM prediction**
ln (median load F)	12.008	11.877
ln (std. dev. load F)	0.556	0.554
ln (median load f)	11.423	11.521
ln (std. dev. load f)	0.589	0.608

Supplementary Table 3: **Spore removal kinetics.** Spore removal due to grooming modeled by a Type II functional response model (Type II FRM), mathematically equivalent to a Michaelis-Menten reaction kinetics, with parameters v, k. Values of v and K, reported in the upper left corner of the table, were estimated by minimizing the squared error in statistics (median, standard deviation) between two pairs of distributions: the initially-applied spore load by F- and f-treatment (quantified directly following application of the F- or f-dose by ddPCR; $n = 30$ each), whose statistics are reported in the second column; and the initial spore load distributions back-computed using the fitted values for the Type II FRM for F- and f-ants, whose statistics are reported in the third column.

Supplementary Table 4

Item	Manufacturer	Distributor	Order number
glass covers Ø51.5 mm	Edmund Optics GmbH	Edmund Optics GmbH	44063
petri dishes Ø 35 mm	SPL Life Sciences	Bartelt	11035
petri dishes Ø 60 mm	SPL Life Sciences	Bartelt	11060
UI-1640LE USB 2.0 camera	IDS	IDS	AB.0010.1.28000.00
fixed focal length lens 6MM	Edmund Optics GmbH	Edmund Optics GmbH	67709
Triton X-100	Sigma-Aldrich	Sigma-Aldrich	X100
TissueLyser II	Qiagen	Qiagen	85300
2.8 mm ceramic beads	MO BIO	VWR	MOBO13114-325
1 mm zirconia beads	BioSpec Products	Lactan Chemikalien u Laborgeräte VertriebsgesmbH & Co KG	N0381
425-600 µm glass beads	Sigma-Aldrich	Sigma-Aldrich	G8772
DNeasy96 Blood and Tissue Kit	Qiagen	Qiagen	69506
EcoRI-HF	New England Biolabs	New England Biolabs	R3101
HindIII-HF	New England Biolabs	New England Biolabs	R3104
2xddPCR Supermix for probes	Bio-Rad	Bio-Rad	1863010
nuclease-free water	Sigma-Aldrich	Sigma-Aldrich	W4502
QX200 droplet generator	Bio-Rad	Bio-Rad	17005227
96-well plates	eppendorf	eppendorf	0030128575
T100 Thermal Cycler	Bio-Rad	Bio-Rad	1861096
QX200 droplet reader	Bio-Rad	Bio-Rad	1864003
Sabouraud-4% Dextrose Agar	Sigma-Aldrich	Sigma-Aldrich	84088

Supplementary Table 4: **Ordering details for materials used in this study.** For each item, the manufacturer, distributor and order number are provided.

Supplementary Figure 1

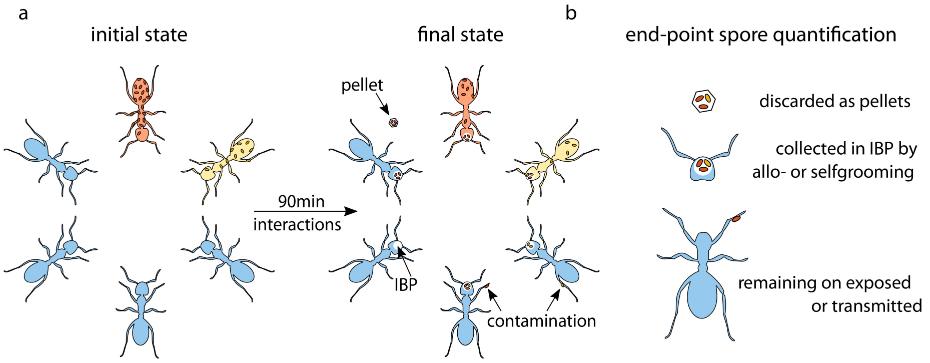

Supplementary Figure 1: **Quantification of fungal spore removal and transfer.** Conceptual depiction of the initial experimental treatment of two individuals in a group of six ants (exemplified in an Ff replicate; F red, f yellow), followed by spore removal and transmission through the ants' individual and social hygiene behaviors, which we determined by spore quantification of each individual after the end of the experiment. **(a)** In each group of six ants (99 replicates), two ants were treated (see Fig. 1a), and four nestmates (N, blue) remained untreated. In all replicates containing two pathogen-treated individuals (treatment groups FF, Ff, ff), each ant received one of the two distinctively labelled spores, i.e. either the GFP- or RFP-variant of the same *M. robertsii* strain (randomized between replicates). In groups with one pathogen-treated and one control-treated individual (FC, fC), the spore-treated individual received one of the fluorescent variants (randomly assigned 50%-50% across replicates). Over the course of the 90 min of the post-treatment phase, self- and allogrooming leads to a reduction of the spore load on the spore-treated individuals, an accumulation of spores inside the infrabuccal pocket (IBP) in the head of the grooming ants, and can lead to contamination of their body surface. Spores collected, compacted and disinfected in the IBP are expelled as pellets into the nest. **(b)** Absolute numbers of both GFP and RFP-labelled spores were quantified after the end of the experiment, separately for the head and body of each ant. The total number of spores disgorged by the ants as pellets was also quantified for each replicate, total $n = 1265$ multiplex ddPCR samples (594 head, 594 body samples and 77 pellet pools). The number of spores retrieved from the ant heads and pellets were used to assess pathogen removal by self- and allogrooming. Spores quantified from the body samples of spore-treated ants reflect both, the remaining spore load of non-removed spores from the initially-applied F- of f-treatment (same-label spores of original treatment), and transmission from the other spore-treated individual (differently-labelled spores). Spores quantified from the body samples of the untreated nestmates (sum of RFP- and GFP-labeled spores) reflect contamination occurring during sanitary care.

Supplementary Figure 2

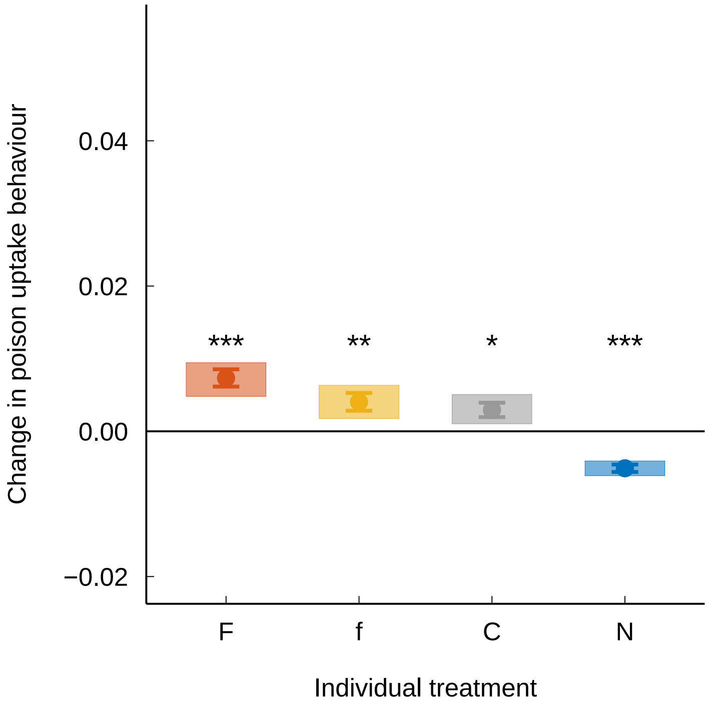

Supplementary Figure 2: **Treatment-induced changes in poison uptake behavior.** Difference in the effective time that the ants spent in the post- vs pre-treatment period taking up their antimicrobial poison from the opening of their poison gland (acidopore) into their mouth, thereby achieving chemical disinfection. All treated ants, i.e. the individuals having received a high (F, red) or low spore load (f, yellow), but also the control-treated individuals (C, gray) increased their poison uptake behavior in the post-period. Instead, the untreated nestmates (N, blue) reduced their poison uptake behavior after treatment of their group members. Mean±sem and 95% CI depicted per ant treatment ($n = 594$ ants from 99 replicates; Suppl. Tab. 1). Significance of paired Wilcoxon tests post- vs pre-treatment period: two-sided p-values, adjusted for multiple testing, as analysed in combination with the other behaviors reported in Suppl. Tab. 2., F, N: $p \leq 1e^{-11}$ (depicted by ***), f: p = 0.0047 (depicted by **), C: p = 0.0158 (depicted by *). Source data are provided as a Source Data file.

Supplementary Figure 3

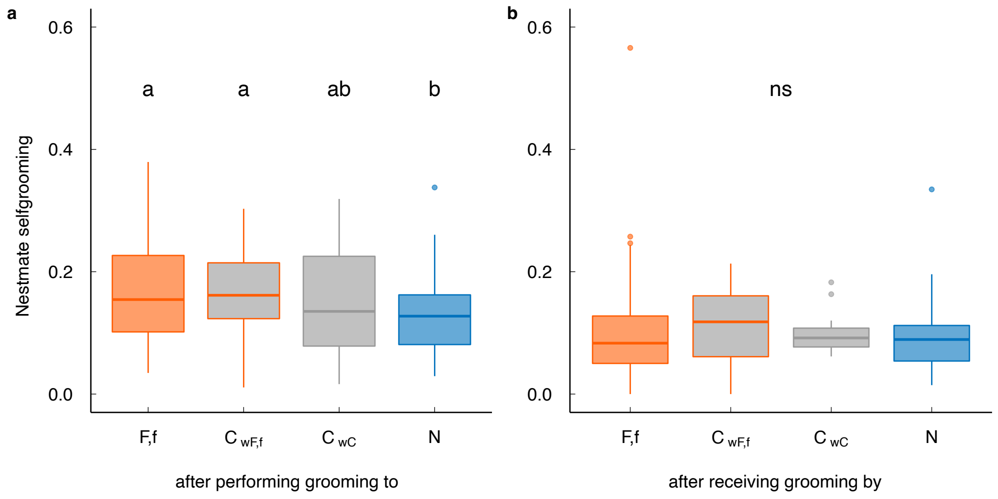

Supplementary Figure 3: **Nestmate selfgrooming following performed or received allogrooming.** Time that nestmates spent selfgrooming their head and body after **(a)** having performed grooming to, or **(b)** having received grooming by group members, depending on the treatment of the latter (box color: spore-loaded individuals F or f, orange; control-treated C, gray; nestmate N, blue). C-individuals were considered separately for when they were paired with a spore-treated ($C_{wF,f}$) individual (orange outlines), or a second C-individual (C_{wC}; gray outlines), i.e. in a group that contained spores or was pathogen-free. **(a)** Nestmates selfgroomed more after having groomed a spore-treated individual than after having groomed another nestmate; they also selfgroomed more after grooming a C-individual, yet only if the latter was paired with another spore-treated, but not another control-treated individual (Kruskal-Wallis Test χ^2 = 15.00, $df = 3$, two-sided $p = 0.002$; $\eta^2 = 0.053$; based on the selfgrooming behavior of N-individuals within 3 minutes after performing grooming towards group members or until their next allogrooming event (F,f, $n = 82$; $C_{wF,f}$, $n = 33$; C_{wC}, $n = 17$; N, $n = 99$). Different letters indicate significant posthoc comparisons at significance level $\alpha < 0.05$; two-sided $p-$values; F,f–N: $p = 0.003$; $C_{wF,f}$–N: $p = 0.017$, all others n.s.). **(b)** The selfgrooming performed by nestmates did not depend on the treatment of the individual that performed grooming to it (Kruskal-Wallis Test $\chi^2 = 2.256$, $df = 3$, two-sided $p = 0.521$; $\eta^2 = -0.004$; F,f, $n = 66$; $C_{wF,f}$, $n = 23$; C_{wC}, $n = 14$; N, $n = 99$; same time window as above). Boxplots in a,b show median (horizontal line), interquartile range (box), with whiskers representing the range of data falling within 1.5-times the interquartile range above the upper and below the lower quartile; data points outside this range plotted individually. All $p-$values adjusted for multiple testing. Source data are provided as a Source Data file.

Supplementary Figure 4

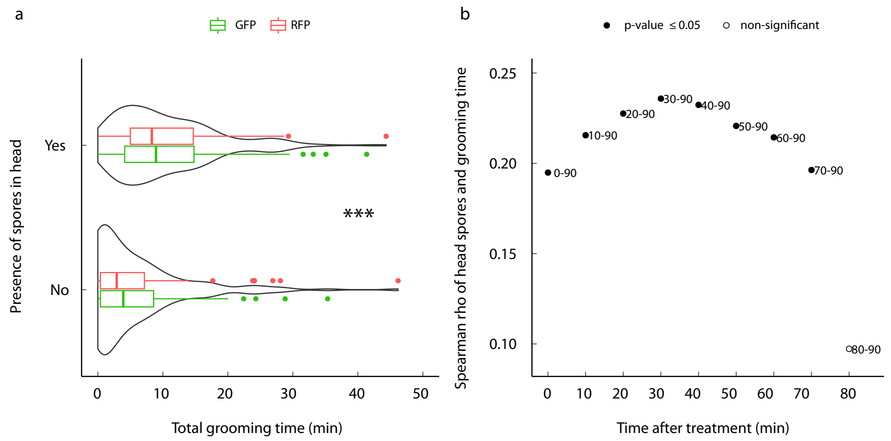

Supplementary Figure 4: **Relationship between performed grooming and head-sampled spores.** **(a)** The total time a nestmate (N) had spent grooming a spore-treated individual predicted the presence/absence of the matching spores in its head at the end of the experiment, independent of spore label (GFP, green; RFP, pink; $n = 524$ data points from 328 N from the 82 groups except CC; Suppl. Tab. 1), showing that ants which groomed the treated individual for a shorter period had a lower probability of having the respective spores in their head, and vice versa (Logistic regression, LR $\chi^2 = 46.5$, $df = 1$, two-sided p-value: $p = 1.61e^{-6}$, depicted by ***). Boxplots show median (line), interquartile range (IR, box), 1.5-times IR (whiskers) and data points outside this range. **(b)** There is a non-linear dependence between the spore number found in the nestmates' heads at the end of the experiment and the duration of grooming they performed towards the treated ant with matching spores. The strength of this relationship (Spearman rho) depends on the observation period. In the last sixty minutes of the experiment (interval 30-90 min) the correlation is highest, and weakens as we consider shorter periods of time (i.e. the last 50, 40, 30 and 20 min). The grooming activity of a nestmate in the last 10 minutes of the experiment (interval 80-90 min) is no longer correlated to the spores in its head, likely due to the very little grooming occurring during this period. Also when looking back for periods longer than 60 minutes the correlation weakens, suggesting that the spores collected during the early grooming events were already disgorged as pellets, and no longer present in the head at the end of the experiment (Spearman-rank correlation between spore number in the head and the grooming performed towards the ant with the corresponding spores, at a given time-interval; rho values labeled with their respective interval bounds, two-sided significant correlations at $\alpha < 0.05$ after correction for multiple testing depicted by filled symbols, non-significant by empty symbol). Source data are provided as a Source Data file.

Supplementary Figure 5

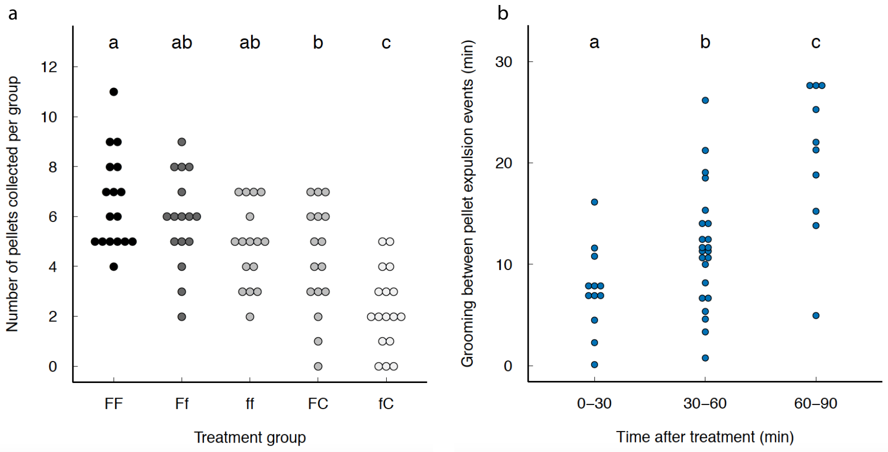

Supplementary Figure 5: **Pellet formation depending on group treatment and experimental period. (a)** The number of pellets that a group of six ants containing at least one pathogen-exposed individual (FF, Ff, ff, FC, fC; $n = 82$ replicates) produced was significantly predicted by group treatment, and hence overall applied spore dose (as shown in Fig. 1a and indicated here by intensity of gray of the replicate data points). Most pellets were produced in groups with two highly spore-treated ants (FF) and the least in groups containing only a single low-level treated ant (fC; LM, $\chi^2 = 186.72$, $df = 4$, two-sided $p = 3.77e^{-11}$; $\eta^2 = 0.409$, $\omega^2 = 0.376$; letters denote significant two-sided post hoc differences at $\alpha \leq 0.05$: FF-ff and Ff-FC: $p = 0.014$, FF-FC: $p = 0.0007$, FF-fC: $p = 2.21e^{-8}$, Ff-fC: $p = 1.84e^{-6}$, FC-fC: $p = 0.011$, ff-fC: $p = 0.0005$, all others n.s.). CC treatment replicates were not included, since none produced any pellets. Note that, with an average number of $1.7\text{x}10^3$ spores, pellet size was independent of treatment group (Kruskal-Wallis Test $\chi^2 = 4.57$, $df = 4$, $p = 0.335$). **(b)** We determined the duration of grooming performed by the nestmates (N, blue) towards the spore-treated individuals (of the matching spore label, see Suppl. Fig. 4) between consecutive observed pellet expulsions ($n = 45$ 2nd to 4th pellets expulsed by N-workers across treatments). The grooming time between pellet expulsions increased over the course of the experiment (Kruskal-Wallis test between first, second and third 30 minute-periods of the experiment: $\chi^2 = 14.92$, $df = 2$, two-sided $p = 0.0006$, $\epsilon^2 = 0.339$; letters denote significant two-sided post hoc differences at $\alpha \leq 0.05$: period 1-2: p = 0.041, 2-3: $p = 0.004$, 1-3: $p = 0.002$). This indicates that a nestmate took longer to reach the filling state triggering pellet expulsion for the same grooming effort, the lower the current spore load of the groomed ant (Fig. 2b), revealing decreasing spore removal efficiency with decreasing spore load (Type II functional response model; Suppl. Tab. 3). $P-$values adjusted for multiple testing.Source data are provided as a Source Data file.

Supplementary Figure 6

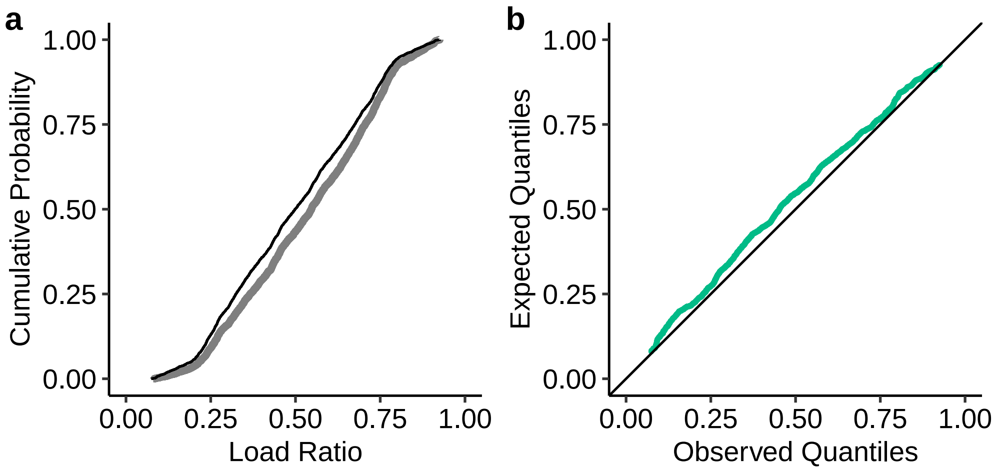

Supplementary Figure 6: **Spore load on the groomed individual** out of total spores on both spore-treated ants, **(a)** shown by the empirical (grey bold) and expected (black thin) Cumulative Distribution Functions (CDFs), and **(b)** as probability (quantile-quantile, QQ) plot (green; guide line in black). Based on n = 196 N from the 49 FF, Ff, ff replicates; same data as in Fig. 2c. Source data are provided as a Source Data file.

Supplementary Figure 7

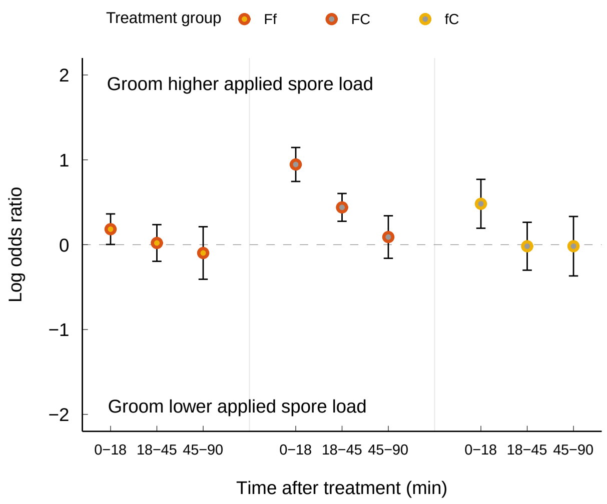

Supplementary Figure 7: **Change in nestmate preference to groom the individual with the higher applied spore treatment over the course of the experiment.** The log odds ratios that the nestmates choose the individual with the higher initially-applied spore load (F in Ff, F in FC, f in fC) given for the groups where the two treated ants had received different initial treatment (Ff, FC, fC; $n = 4823$ grooming events by 196 N from 49 replicates). The log odds ratios were calculated separately for the early, mid and late periods of the experiment each containing a similar number of grooming events (leading to unequal time intervals as most grooming is performed at the beginning of the experiment). Mean±sem per treatment group and period shown; dotted line depicts log odds ratio of zero, which is the absence of any preference between the two treated ants. Early in the experiment the ants show a strong preference to target the individual that was initially exposed to pathogenic spores (F, f) over control-treated ants. At this early period, they also choose between two spore-treated individuals, preferentially targeting the individual treated with the higher applied spore dose (F over f). With time, this preference towards the individual with the higher initially-applied spore load becomes moderate or undetectable. Initially-applied spore loads also do not reliably predict the spore load differences between the treated ants throughout the experiment, as their loads dynamically change with the grooming they receive (Fig. 2b). In conjunction with Fig. 6a and Suppl. Fig. 9, this reveals that the ants react to the current spore load differences between the two ants, whereas the difference in the initially-applied spore loads is relevant only in the early stages of the experiment, where applied spore load still approximates current spore load. Source data are provided as a Source Data file.

Supplementary Figure 8

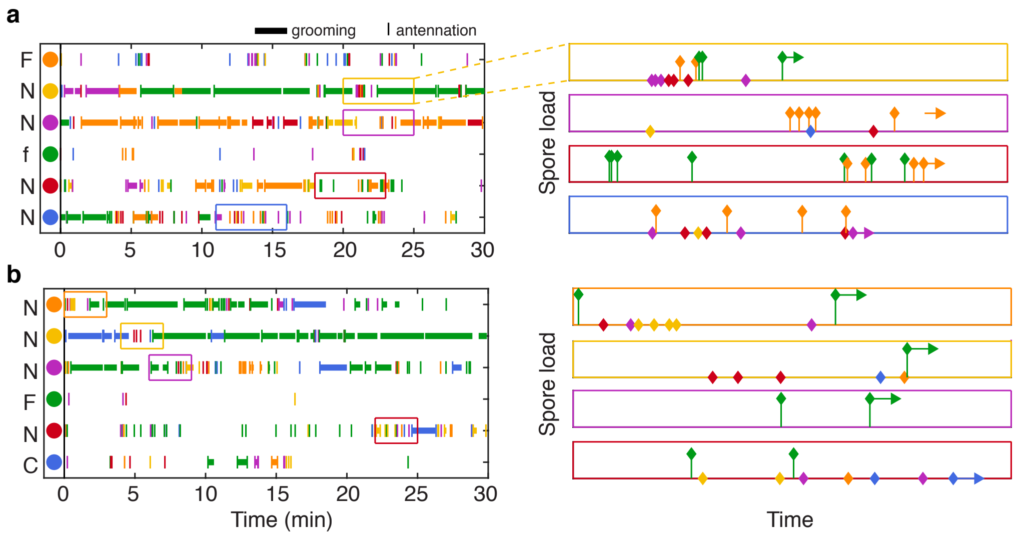

Supplementary Figure 8: **Sequences of antennation events that precede grooming decisions are candidates for probing postulated by the sequential-choice rule.** Detailed observation of antennation (vertical lines) and grooming (horizontal thick lines) behavior performed by each ant (shown by its individual color-code and treatment: F high, f low spore-load treated, C control-treated individual, and N nestmate) during the 30 min after treatment for **(a)** an Ff and **(b)** an FC example; receiving ant being indicated by line color. Boxes with a zoom-in at right outline one antennation series per nestmate. In the zoom-ins, the current spore load of the receiving ant is shown by diamond height (zero spore load for C and N individuals). Shown are all antennations (diamonds) that precede a grooming event (arrow; box color identifies performing nestmate individual). Ants typically antennate several different individuals of differing spore load before grooming. A grooming event often directly follows an antennation (arrows starting from diamond), and frequently targets one of the spore-treated individuals. Source data are provided as a Source Data file.

Supplementary Figure 9

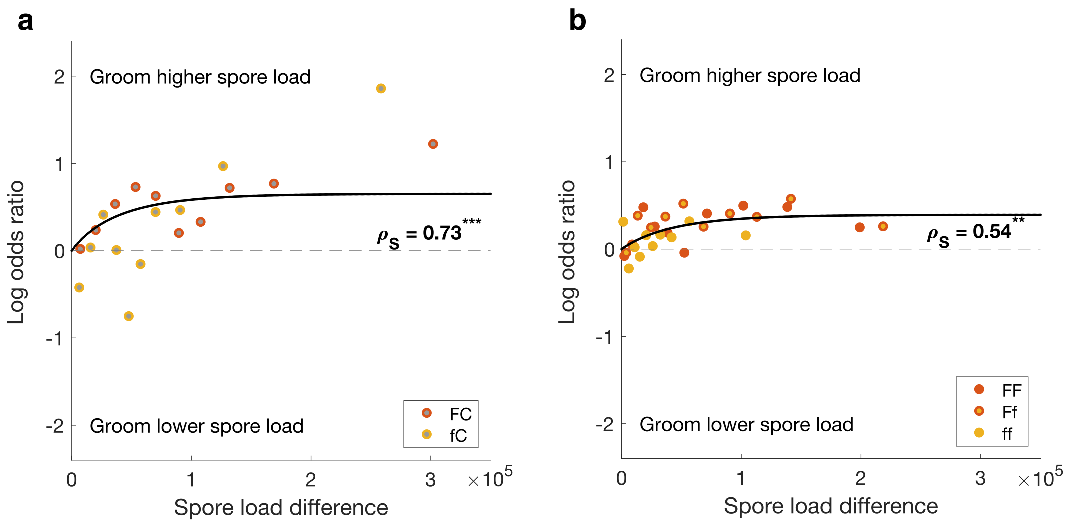

Supplementary Figure 9: **Nestmate preference to groom the individual with the higher current spore load depending on load difference.** We consider the grooming events performed by the nestmates (N) towards each of the two treated individuals, and present the log odds ratio to groom the individual with the higher current spore load of the two, in relation to their spore load difference (data fairly binned into ten categories by spore load difference between the treated ants), shown separately for groups with **(a)** one spore-treated and one control-treated individual (FC, fC; $n = 3128$ grooming events from 132 N from 33 replicates) and **(b)** two spore-treated individuals (FF, Ff, ff; $n = 5001$ grooming events from 196 N from 49 replicates). Nestmates did not only preferentially groom the spore-treated individual over the control-treated individual (a; Spearman-rank correlation $S = 356$, two-sided $p = 0.0004$, depicted by ***) but also preferentially targeted the currently higher-loaded of two spore-treated individuals (b; Spearman-rank correlation $S = 2077.7$, two-sided $p = 0.002$, depicted by **). Therefore, the observed overall grooming preference towards the individual with the currently higher spore load (Fig. 6a) is not only explained by ants being able to differentiate between a pathogenic and non-pathogenic treatment, but also their capacity to preferentially groom the higher spore-load from two pathogen-treated individuals (see also Fig. 2c). Spearman rho given for a,b. Black line depicts the theoretical prediction (in Suppl. Note 3, Eq.10). Source data are provided as a Source Data file.

Supplementary Figure 10

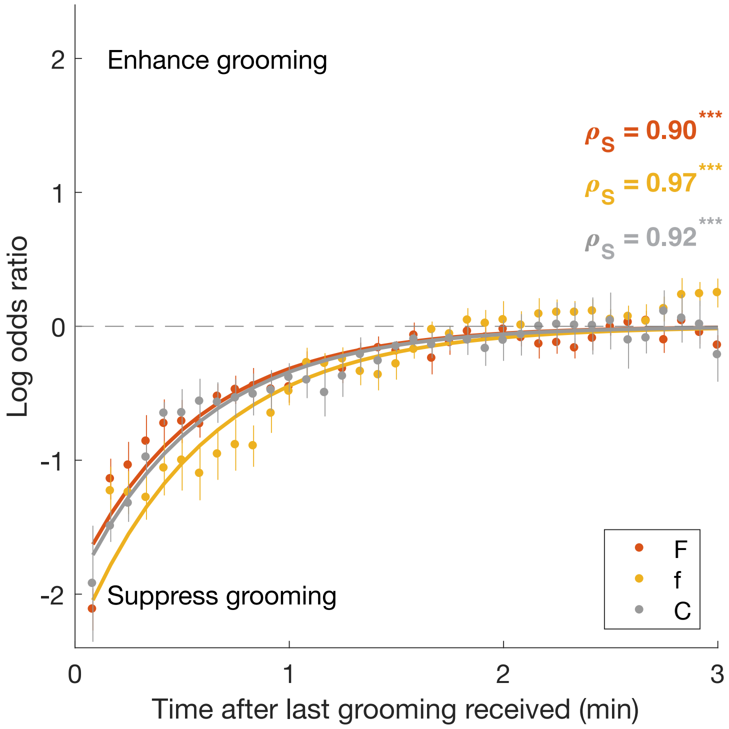

Supplementary Figure 10: **Effect of social feedback on grooming performance of the treated ants.** The log-odds ratio (LOR) to perform allogrooming as a function of time since last received grooming by others, computed separately for the ants treated with a with a high (F; red, $n = 66$) and a low (f; yellow; $n = 65$) spore load, as well as for the control-treated ants (C; grey; $n = 33$), reveals that all treated ants, independent of their spore load, are suppressed to perform own grooming shortly after having received allogrooming by others. The LOR is based on the relative count of the performed grooming events following after received grooming to the performed grooming events followed after non-receiving, each properly rescaled and as a function of the elapsed time (in min) between the time bins of interest. Time-dependence shown by Spearman rank correlation (mean±std over 5s bins; solid lines display exponential fits, *** denotes two-sided significance of $p < 0.001$, namely $p < 1e^{-11}$). Relates to Fig. 6b, where all treated ants are pooled, as they show similar LOR. Analysis based on $n = 99$ replicates. Source data are provided as a Source Data file.

Supplementary Figure 11

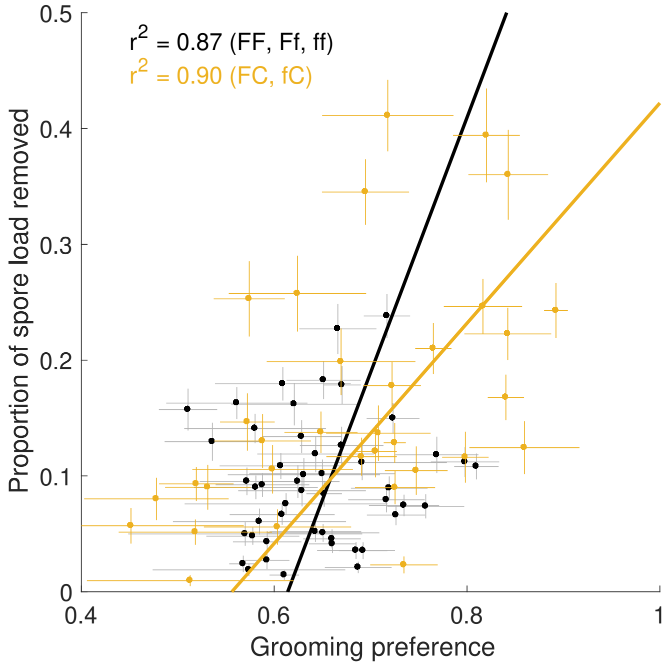

Supplementary Figure 11: **Spore removal effciency by grooming vs. grooming preference towards the currently higher-load individual.** The proportion of spores removed by nestmate allogrooming and pellet formation (from the experiments) correlates to the grooming preference towards the currently higher-load individual in the group, i.e. the ratio of the performed grooming bouts towards the treated individual with the higher current spore load compared to all grooming bouts towards both treated ants (from the five replicate simulations). The analysis was done by fitting the linear functional model for groups with two pathogen-treated individuals (FF, Ff, ff; black; $n = 49$ replicates) or for groups with only a single pathogen-treated and one sham-treated individual (FC, fC; yellow; $n = 33$ replicates). R-squared of the fit is given. Same data as in Fig. 7a, where data were not separated for the groups containing only one or two pathogen-treated individuals ($n = 82$ replicates). Source data are provided as a Source Data file.

Supplementary Note 1: Model of collective spore removal activity of ants

Model outline

We start by outlining a basic model of spore removal activity of ants. This section provides a short overview of the modeling components without explaining their details. Each part of the model, along with model selection, validation, and assessment of the performance of the best model is explained in detail in the following sections.

At any moment an ant can be classified according to its spore removal activity into one of the three behavioral states: allogrooming (A), selfgrooming (S), and inactivity (X). While the states S and X refer to a single ant, allogrooming captures interaction between a pair of ants, one grooming (in state A), the other being groomed (in any state). We did not include the poison uptake behavior and the pellet disgorgement, although these behaviours were also observed, as they are not spore removal behaviours. The state of the system at any moment can be described by the state of each ant, the nature of interactions (who is grooming who) and by an additional variable of perturbation load ℓ for each ant, which captures the level of perturbation of the focal ant due to initial treatment (exposure to pathogen, treatment by sham solution, etc.). The stochastic model of spore removal behavior of ants can be summarized into the following components:

1. Stochastic transitions between behavioral states. The model, in contrast to the traditional models of collective behavior in social insects, aims at capturing decision making involved in spore removal. Decisions are captured by stimulus-dependent and activity-dependent transition rates between the individual behavioral states. Given two grooming states (A, S) and one inactive state (X) there are eight possible transition types between them: $A \to A$, $A \to S$, $A \to X$, $S \to A$, $S \to S$, $S \to X$, $X \to A$, and $X \to S$, as shown in Fig. 3a (of the main text). Each of these rates depends on a couple of decision variables, which capture the recent activity and the recent sensory inputs of the focal ant. Transition rate dependence on these variables is formulated using the Generalized Linear Model (GLM) framework, which allows simple inference of rates from experimental data and comparison of models using information-theoretic quantities. Good choice of the variables is crucial for the accuracy of the model as argued in the study of the theoretical framework for the general class of collective behavior models [1]. Moreover, the proposed modeling framework allows for modularity: each individual in this framework can have a different set of behavioral states, stochastic transitions between different behavioral states may depend on a different set of available decision variables, etc. Nevertheless, the transition rates in such a collective behavioral model can be uniquely inferred from the data and

systematically compared to models with different modular components (set of behavioral states, set of decision variables, etc.).

2. Decision variables in the model. The transition rates of the focal ant are modulated by a couple of decision variables, which integrate signals from its own past behavior, from other individuals, or from the environment. In the post-phase (i.e. the second phase of the experiment after ant treatment, duration 90 min) the baseline decision variables are: recently performed allogrooming y_{P}, recently received allogrooming y_{R}, considered also for the pre-phase (i.e. the first phase of the experiment before ant treatment, duration 30 min). In addition, we use a decision variable, which captures decision making in response to a pathogenic or non-pathogenic initial perturbation (or no perturbation at all). We introduce the term "perturbation load" to measure the level of this initial perturbation (for the pathogen-treated ants it is equal to the number of pathogen spores the ant was initially contaminated by). The corresponding decision variable is defined as the last seen nonzero perturbation load during allogrooming y_{La} within a time interval of interest (or during both allo- anf selfgrooming y_{Las}, for detailed definition see Suppl. Tab. 5). Each of the variables y_{P}, y_{R}, y_{La}, and y_{Las} captures spore removal behavior in a time interval before present of length Δ_{P}, Δ_{R}, Δ_{La}, and Δ_{Las}, respectively, and thus it cannot be described solely using the state variables in the present time. The definitions of the memory parameters Δ_{P}, Δ_{R}, Δ_{La}, and Δ_{Las} are summarized in Suppl. Tab. 5.

3. Type II FRM dynamics of the perturbation load. While the previous two components of the model are integral parts of the GLM framework for decision making (in this case of ants perturbed by the pathogen exposure), the dynamics of perturbation load comes from experimental observation. Spore abundant systems show a large rate of spore removal, limited by the size of the group and by the maximum speed of removal of each ant, i.e., the removal is linear in time. However, when the spore abundance is low the removal is exponential due to rate encounter of spores by grooming ants. These two observations are integrated in one of the simplest and yet broadly applicable dynamical models of decay (broadly applicable across various fields, such as ecology, chemical kinetics, etc.), i.e., the Type II functional response model.

4. Grooming preference. Experimental observations indicate that grooming preference is modulated by the perturbation load. Thus, the model considers three alternatives for the choice of recipient of allogrooming

   - *Uniform random sampling* (URAND): The ant, which receives allogrooming by the focal ant, is chosen randomly from all nonfocal ants with equal probability. This is a null model, which will be used for comparison with

other models where grooming depends on the amount of pathogenic or non-pathogenic perturbation.

- *Maximum sampling* (MAX): The ant, which receives allogrooming is the nonfocal ant with the largest nonzero detectable load if at least one such ant is present, otherwise uniform random sampling applies.
- *Sequential load-biased sampling* (SEQ): The ant to receive grooming is chosen by a sequential rule. Sample an ant and accept probabilistically according to a load-dependent acceptance probability. If not accepted, move to a next random non-focal ant and repeat until an ant is picked.

5. Initialization of perturbation load. The quantity $\ell_k(0) > 0$ equals the initial number of spores for each ant k that has been exposed to a pathogen. C ants (with no spores) have initial perturbation load set to $\ell_k(0) = L_\mathrm{C} > 0$ (Suppl. Tab. 5), nestmates satisfy $\ell_k(0) = 0$.

6. Suppression of activity of the treated ants. The transition rate $X \to A$ of the treated ants (F, f, C) equals the transition rate of the nestmates (N) divided by a factor ρ (see Suppl. Tab. 5). If $\rho = 1$ the transition model is blind to the type of the ant. The factor ρ thus accounts for the reduced allogrooming activity of all treated ants.

The model can be inferred separately for the pre- and post-phase, although the main focus here is the post-phase. The rules 1-2 define a theoretical GLM model of decision making, which can be fit using the experimental data. Although the model setup is straightforward, the greatest challenge is to find suitable decision variables, which not only fit the existing data, but which also have a predictive power. Next, the rules 3-4 are necessary to fully specify the forward model. Forward stochastic simulation of such a model returns data with the same level of detail as our experimental recordings. The rules 3-4 represent the biological insight about the system. Finally, rules 5-6 are the necessary modifications of the model, required to obtain a good match with the empirical data. The optimal model is found using a systematic model selection based on log-likelihood of the inferred probabilistic model and on the error in the activity traces from the stochastic simulations.

Model setup

Here we describe a stochastic dynamical model to explain the spore removal behavior of ants, observed in the experiments. Each dish in the experiment contained 6 ants, whose behavior was first recorded for 30 mins without any perturbation (pre-phase). Two ants were then exposed to different levels of pathogen, which resulted in four types of ants in the post-phase experiments (duration 90 mins). We aim to construct a single

model that explains the behavior of all ants without needing to split the inference to the ant types: F, f, C, N, according to the pathogen dose (high, low, control, nestmates). The ants in the pre-phase are identical in terms of the treatment (no treatment, all nestmates). There are three key behavioral states: S - selfgrooming, A - allogrooming, X - idle state, see Fig. 3a for the schematic representation of the transition model. The S state includes head and body selfgrooming. We made this choice for the sake of model simplicity and tractability (rather than modelling the two selfgrooming states, head and body selfgrooming, separately). If the model S state included just body selfgrooming, it could replicate more closely the spore-removal dynamics, which has been calibrated from data using the Type II response model using body selfgrooming alone; as a tradeoff to that choice, however, we would completely neglect head selfgrooming as a behavior that is actually more frequent than body selfgrooming, leading to a significantly biased estimation of behavior-switching rates. Instead, we chose to lump the two biologically distinct selfgrooming behaviors into the single model S state. This ensured a consistent estimation of behavior-switching rates, while inducing some imprecision in the spore removal dynamics. This imprecision, however, is limited, because the spore removal is dominated by allo- (and not self-)grooming events. The state A summarizes all observed allogrooming events. The scheme in Fig. 3a (of the main text) therefore represents the core of the ant behavior model. It considers 8 possible transitions between the three states $x \in \{A, X, S\}$. These transitions are:

$$X \to S,\ S \to X,\ A \to X,\ S \to S, X \to A,\ S \to A,\ A \to S,\ A \to A\,. \qquad \text{(Eq.1)}$$

The state X is the default inactivity state and it can only be terminated by an onset of allo- or selfgrooming. Thus, the transitions $X \to X$ are not considered in the model. On the other hand, the transitions from $S \to S$ may happen if the same ant switches between different types of selfgrooming (head or body selfgrooming). Similarly, the $A \to A$ transitions may occur when the same ant switches the target of its allogrooming to another ant. We proceed by systematically building a set of models from simple to more complicated forms. From the modeling perspective we assume that all ants follow identical behavioral principles, apart from randomness, which is inherent to switching between these behavioral states.

Transitions between the behavioral states (x – outgoing state, x' – incoming state of the focal ant) are random and the transition rates account for perceived external stimuli (i.e. sensory cues) or internal information (i.e. level of recent activity) of the focal ant $\phi_i(y)$, which are modulating the weights α_i through the following relationship

$$\lambda(x \to x', y) = \exp\left(\sum_i \alpha_i(x \to x')\phi_i(y)\right)\,. \qquad \text{(Eq.2)}$$

For ϕ_i we use the tiling functions on the space of the values of y, called decision variables. For instance, if y represents the level of activity of the focal ant (as shown later)

three activity levels can be considered: no activity, low activity and high activity. At each time only one summand in (Eq.2) is present – the one with an index corresponding to the current activity level y of the focal ant. The decomposition in (Eq.2) corresponds to discretizing the possibly continuous decision variable y into a finite number of bins and using different transition rates for different bins. If the decision variables only depend on current time, and do not depend on the system's history, the model has a Markovian property and the rates are memoryless. However, we allow the variables y to be time-averaged quantities, capturing memory-based decision making of the ants.

The contamination of an ant by a pathogen is introduced into a model through a new virtual variable ℓ_k, which is a proxy for the magnitude of the perturbation by spore contamination or the perturbation by the application of a sham-treatment of the focal k-th ant at a given time. For example, perturbation load for any nestmate is initially zero and it stays zero at all times. Next, perturbation load of a pathogen-exposed ant is initialized using the empirical data, i.e., the number of actual spores the ant was contaminated by. Thus the perturbation load for the pathogen-exposed ants is a proxy for the dynamically-changing and hence experimentally unobservable (but determined by the Type II FRM kinetics) current spore load of the ant at any time of the experiment. Finally, perturbation load of an ant exposed to a non-pathogenic solution reflects the amount of perturbation by treatment per se and thus is nonzero, even though the ant is not contaminated by pathogen. Note that the ants treated by a non-pathogenic solution are the correct control for evaluation of the impact of pathogen contamination, as opposed to the nestmates, which are not perturbed.

In an agreement with biological intuition we assume that the perturbation load decays when the ant is groomed (both allo and self) and stays constant in the absence of grooming. Empirical evidence suggests Type II FRM (or Michaelis-Menten) kinetics for the decay of the spore load

$$\frac{d\ell_k}{dt} = \begin{cases} -v\frac{\ell_k}{\ell_k+K}n_k & \text{if the } k\text{-th ant is groomed at time } t \\ 0 & \text{else} \end{cases} \qquad \text{(Eq.3)}$$

where grooming includes both allo- and selfgrooming. The factor n_k stands for the number of ants at a given time, which groom the focal ant (including self). Thus, when the ant is being groomed by multiple ants the decay rate on the right hand side of (Eq.3) is larger by the integer multiple n_k. As this part of the supporting information is devoted to the mathematical modeling of the spore removal behavior, we will dominantly use the theoretical concept of the perturbation load and for brevity we will refer to it as load (without writing the adjective perturbation).

The key for obtaining a successful model of social spore removal behavior of ants is to choose a suitable set of decision variables. The most logical candidates, based on empirical data, are based on the recent activity (performed and received grooming) and the perturbation load recently encountered during grooming. Since grooming can be of

Supplementary Table 5: Optimal parameters and notation used in the model.

Notation	Value	Fixed parameters of the model
v	5.22	Maximum rate of decay in the Type II FRM for spore load decay
K	$50,119$	Amount of spore load at which the rate of decay is half of the maximum in Type II FRM
L_{C}	5×10^4	Initial perturbation load of C ants
L_0	5×10^4	Load detection threshold

Notation	Value	Inferred parameters of the model
Δ_{P}	2m	Memory in the performed allogrooming, inferred from the P model
Δ_{R}	1s	Memory in the received allogrooming, inferred from the R model
Δ_{La}	2m	Memory in the load seen during allogrooming, inferred from the La model
Δ_{Las}	30s	Memory in the load seen during any allo- or selfgrooming, inferred from the Las model
ρ		The suppression of the transition rate $X \to A$ for the treated ants compared to nestmates (model dependent)

Notation		Variables in the model
$\ell_k(t)$		Perturbation load of the ant k at time t
$x \in \{\mathrm{S}, \mathrm{A}, \mathrm{X}\}$		Behavioral states: selfgrooming, allogrooming, idle state
P (R)		Model with transition rates dependent on the recent performed (received) activity
La (Las)		Model with transition rates dependent on the recent perturbation load seen during allogrooming (allo + self)
y_{P}		Fraction of the last Δ_P seconds when the focal ant allogroomed others
y_{R}		Fraction of the last Δ_R seconds when the focal ant received allogrooming
y_{La} (y_{Las})		Last nonzero perturbation load experienced by the focal ant during allogrooming (allo + self) in the previous Δ_L seconds (0 if no nonzero load was seen)

two types (allo and self) one may distinguish three versions of the grooming considered: allo, self, and both allo+self. Similarly, the perturbation load, which the ants observe while grooming, can be of the same three types: observed during allo, self, and allo+self grooming. All such variations of our analyses were considered but for simplicity we only present a reduced set leading to the most informative decision variables. These are

denoted by the subscripts y_{P}, y_{R}, y_{La}, and y_{Las}, where for the focal ant k

- $y_{\mathrm{P}}(t)$ – proportion of time in interval $[t - \Delta_{\mathrm{P}}, t)$, during which ant k was allogrooming;
- $y_{\mathrm{R}}(t)$ – proportion of time in interval $[t - \Delta_{\mathrm{R}}, t)$, during which ant k received allogrooming;
- $y_{\mathrm{La}}(t)$ and $y_{\mathrm{Las}}(t)$ – the last nonzero perturbation load an ant k has seen during allogrooming (La) and allogrooming or selfgrooming (Las) within time interval $[t - \Delta_{\mathrm{La}}, t)$ and $[t - \Delta_{\mathrm{Las}}, t)$, respectively;

as summarized in Suppl. Tab. 5. By definition the variable y_{P} is nondimensional and bounded between $[0, 1]$, while the variable y_{R} is bounded between $[0, 5]$, reaching a maximum value only when the ant is simultaneously allogroomed by all the remaining ants during the whole considered time interval of length Δ_{La} or Δ_{Las}.

We consider the following models with zero, one, or two decision variables affecting each transition rate (to avoid overfitting, we do not consider more than two variables):

- **Model with 0 decision variables:** Transition rates $\lambda(x \to x') = c_{x \to x'}$ are constant and depend only on the transition type (C model). There are 8 different fitting parameters, one for each transition type. The transition rates, computed using log-likelihood maximization, are the number of observed transitions of the corresponding type divided by the total amount of time spent at the outgoing state.
- **Models with 1 decision variable:** Transition rates $\lambda(x \to x', y)$ depend on one of the decision variables y_{P}, y_{R}, y_{La}, or y_{Las}. We refer to these models as P, R, La, or Las models.
- **Models with 2 decision variables:** Transition rates $\lambda(x \to x', y_1, y_2)$ depend on either performed or received activity and on the perturbation load (La or Las). The reason for not including a PR model is that the marginal models P, R in principle cannot explain temporal behavior on the long timescales, which, as we shall see, is well explained by the load variable.

In addition, each model can be tested for the whole range of the memory parameter values Δ_* ($*$ stands for the type of the used decision variable). The impact of the choice of Δ_* is explored systematically in order to find the best probabilistic model, i.e. the memory parameter that best explains the data.The optimal values may differ between the models of different complexity.

Model inference

Which stochastic transition model of ant behavior best agrees with the data? This is answered by the log-likelihood function $L(\boldsymbol{\alpha}|\mathcal{D})$, quantifying the evidence the data provide about the parameters $\boldsymbol{\alpha}$ to be inferred. Log-likelihood depends on the states of the system $\mathbf{x}(t)$ and on the decision variables $\mathbf{y}(t)$ in the time window of interest (bold letters represent vector variables, which include separate components for each ant). In particular, it also depends on all the transitions $\mathbf{x}(t_{k-1}) \to \mathbf{x}(t_k)$ between the states at times t_k, indexed by k. Following [1] we have

$$L(\boldsymbol{\alpha}|\mathcal{D}) = \sum_k \left(\ln \lambda(\mathbf{x}(t_{k-1}) \to \mathbf{x}(t_k), \mathbf{y}(t_k)|\boldsymbol{\alpha}) - \sum_{\mathbf{x}'} \int_{t_{k-1}}^{t_k} \lambda(\mathbf{x}(t_{k-1}) \to \mathbf{x}', \mathbf{y}(t)|\boldsymbol{\alpha}) \mathrm{d}t \right) + C \tag{Eq.4}$$

$$= \sum_i \sum_{\mathbf{x},\mathbf{x}'} \alpha_i(\mathbf{x} \to \mathbf{x}', \mathbf{y}_i) \mathcal{N}(\mathbf{x} \to \mathbf{x}', \mathbf{y}_i) - \sum_i \sum_{\mathbf{x},\mathbf{x}'} \exp\left[\alpha_i(\mathbf{x} \to \mathbf{x}', \mathbf{y}_i)\right] \mathcal{T}(\mathbf{x}, \mathbf{y}_i) + C \tag{Eq.5}$$

where $\mathbf{y}_i$ are the centers of the i-th bin corresponding to the i-th tiling function and the included transitions are only those $\mathbf{x} \to \mathbf{x}'$, for which $\mathbf{x}$ and $\mathbf{x}'$ differ only in one component; corresponding to a switch of state of only a single ant at one moment. The equivalent expression in the second line was obtained by rearranging the sums and collecting all transitions and all values for each bin and each transition separately. $\mathcal{N}$ and $\mathcal{T}$ denote the summary statistics, $\mathcal{N}$ collects the total number of transitions for each bin while $\mathcal{T}$ summarizes the total time spent in each state and bin. The function (Eq.5) is concave and thus has a unique maximum. We proceed by finding a set of optimal parameters $\boldsymbol{\alpha}$ by maximizing the function $L(\boldsymbol{\alpha}|\mathcal{D})$ using an optimization tool minFunc in Matlab.

The resolution of the decision variables is reflected in the choice of the tiling functions $\phi_i(y)$ (through the number and width of bins), which in turn influences the number of unknown coefficients $\boldsymbol{\alpha_i}$ to infer. Better resolution of transition rates requires more bins and coefficients α_i, which causes overfitting and low predictive power. We chose three bins for each of the decision variables, sorting them into three categories – negligible, small, large values:

- **Bins for the activity variables P, R:** $[0, 0.01)$, $[0.01, 0.2]$, $(0.2, 5]$ (maximum for the P variable is one, corresponding to allogrooming during the whole interval Δ_P; maximum for the R variable is 5, corresponding to receiving from all other five ants during the whole interval Δ_R).
- **Bins for the load variables La, Las:** $[0, 5\times10^3)$, $[5\times10^3, 3\times10^4]$, $(3\times10^4, \infty]$ (measured in spores).

Despite a crude binning this model showed qualitatively similar results to models with more refined bins while still keeping a small number of parameters.

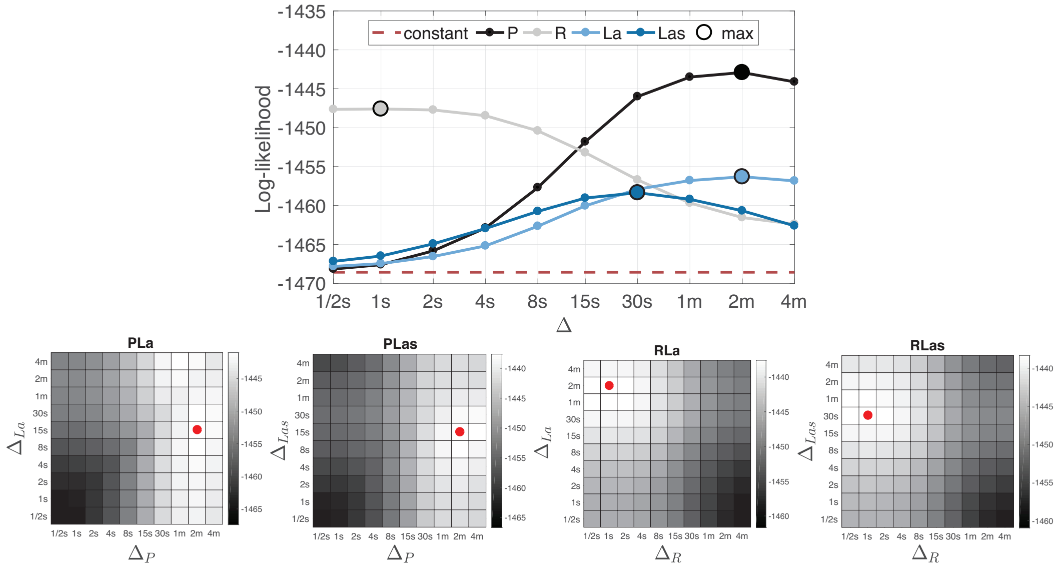

Supplementary Figure 12: Log-likelihood as a function of the memory parameters Δ_{P}, Δ_{R}, and Δ_{L}. The top panel shows dependence of the log-likelihood on memory parameters in models with one decision variable. Maximum is reached for an interior point (given the used discretization). The panels in the bottom show log-likelihood dependence (background shading) on the memory parameters in the models with two decision variables. The global maxima are denoted by the red dots in the centers of the appropriate cells. The results are based on all 99 replicates in Suppl. Tab. 1.

The decision variables of a given model (e.g. PLa model) depend on the current state of the system but also on the behavior during a fixed time interval before present (memory of length Δ_*). The optimal memory for each variable can be systematically inferred from data, based on log-likelihood. Suppl. Fig.12 shows the dependence of log-likelihood on the memory parameters in all considered models with a single decision variable, but also in all considered models with two decision variables (leading to a 2-dimensional dependence). The optima are shown in Suppl. Tab. 6 for all considered one- and two-variable models.

Note that the optimal memory parameters for the models with two decision variables are consistent with the optimal memory parameters for models with a single variable, although in the case of the PLa model it is not the case (even though the log-likelihood function is quite flat and the value corresponding to the combined optimal memory parameters from the single-variable models is close to the global optimum). This means that the two-dimensional optimum is in most cases just a combination of the optima for the one-dimensional models. While in the constant model memory plays no role, the P, R, La, and Las models attain maximal log-likelihood for different memory parameters. The optimal Δ for the P variable is 2 min, for the R variable is 1s, for the La variable is

2 min, and for the Las variable it is 30 s.

Supplementary Table 6: Optimal memory parameters for the variables P, R, and L in all considered models.

Model	P	R	La	Las	PLa	PLas	RLa	RLas
$\Delta_{\text{P/R}}$	2m	1s	—	—	2m	2m	1s	1s
$\Delta_{\text{L/La}}$	—	—	2m	30s	15s	15s	2m	30s

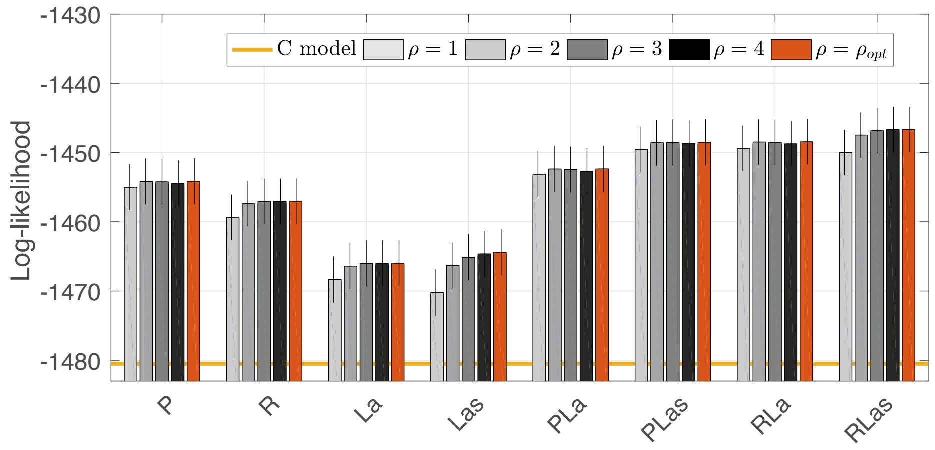

Supplementary Figure 13: Log-likelihood of all considered models (both with one and with two variables). We used all treatments, all replicates and all ants to fit the models. The models also contain a parameter ρ, which is a multiplicator, by which the $X \to A$ transition rates of the treated ants are reduced relative to the corresponding rates for the nestmates. Each model was inferred for the fixed values of $\rho = 1, 2, 3, 4$ (gray bars). In addition, we also jointly inferred the model parameters together with ρ (red bars), returning the optimal value ρ_{opt}. The values of ρ_{opt} ordered left to right as in the figure axis are: $\rho_{\text{opt}} = \{1, 2.27, 3.39, 3.56, 6.17, 2.19, 2.54, 2.31, 4.32\}$. Each log-likelihood, computed for the whole dataset, is complemented by an error bar, computed as a standard deviation of the outcome obtained on a subsampled dataset. The subsampling was realized by leaving out k-th replicate for each treatment for k=1, $2, \ldots, 16$ ($n = 16$ subsamplings). We centered the error bars around the means of the outcomes based on subsampled data (the centers are undistinguishable from log-likelihood for all data). The results are based on all 99 replicates in Suppl. Tab. 1.

Suppl. Fig.13 shows a comparison between the log-likelihood of the models considered. The models incorporate a reduced $X \to A$ transition rates for the treated ants compared to nestmates by a factor of ρ. If $\rho = 1$ the rates for all ants are the same and

the model is blind to the ant type. Among such models model RLa has the highest log-likelihood, closely followed by the model PLas. Naturally, all models with two decision variables perform better than the models with a single decision variable, which in turn perform better than the C model with constant rates.

We explored models where ρ was fixed but different from one, namely, $\rho \in \{1, 2, 3, 4\}$ (gray bars) to see the sensitivity of the model performance on this parameter. Moreover, we studied the model, where ρ was part of the inference (red bars in Suppl. Fig. 13) (note that including factor ρ in the inference does not change concavity of the log-likelihood, it only increases the number of inferred parameters by one). The best performing model (for optimized ρ value) included decision variables R (received allogrooming) and Las (load decays during allogrooming and selfgrooming), closely followed by the models RLa and PLas.

Log-likelihood measures local (in time) performance of the model, i.e., given the history, what is the most likely behavior of the focal ant in the next short time period. Performance of the combined model (e.g. PLa) always outperforms the marginal models (P and La) due to having more degrees of freedom. Suppl. Fig.13 suggests that among models with a single decision variable models P and R have a higher log-likelihood than the L models (where L includes both La and Las models), with model P showing the highest value. However, PL combinations have a lower log-likelihood than RL combinations. This is because large loads La/Las in the dataset typically occur when the performed activity P is large, leading to a high correlation between the recent performed activity (P) and the load seen while allogrooming (or allo+selfgrooming). This correlation may arise in two causal ways: (1) high La/Las may increase transition rates into the active state which in turn increases the P variable; (2) less allogrooming (assuming random recipient) results on average in smaller load seen due to less encounter with the ants with a positive load. The highest log-likelihood is reached by the models, which combine received activity and load variable. We did not include model PR because it cannot capture the long-term response of the system. This is caused by the fact that the momentum in the behavior of the P and R models is dominantly affected by the memory lengths for the P and R activities, which are 2 min. and 1 sec., respectively. On the other hand, the dynamics in the models containing the L-component are driven by the long-term decay of the perturbation load, captured by the Type II FRM kinetics with parameters v and K (in Suppl. Tab. 5). Even if the decay was linear and each contaminated ant was constantly groomed by some other ant (both are exaggerations), it would take more than 10 minutes to remove the initial perturbation load.

Stochastic simulation

Note that the probabilistic transition model describes only the state of the focal ant at a specific time, i.e. its activity or inactivity but not the recipient of allogrooming this ant performs. Knowledge of the rates is thus insufficient for simulating spore removal

behavior of ant communities. To complete the model, we complement it with the rule, prescribing who is chosen as the target of allogrooming.

Fig. 3d (of the main text) shows an overview of the grooming rules considered. They differ by the type of information updating the model requires, ranging from the uniform random pick of the recipient of allogrooming (no information about an ant's current loads used) through rules based on initial perturbation loads of the ants, to rules which require complete knowledge of the current perturbation load of all ants. Despite all these rules are probabilistic they differ by the average amount of perturbation load they remove per unit of time (the MAX rule removes most of the load, SEQ less, and URAND the least amount on average). Even though the experiment performed in our work contained a fixed number of ants in one dish (i.e. six ants, two of which are perturbed) and the rules, which require complete information could be realistic for such a small system, the rule is not feasible for larger groups of ants. However, the sequential rule, where ants with no information acquire information by probing ants one after another, may be realistic even in larger experiments (see Fig. 6 of the main text).

The simulations are performed using a stochastic simulation algorithm (SSA) on the basis of the Gillespie algorithm [2].We do not resolve the motion of the ants in a physical space with encounters due to proximity, only the behavioral response to the stimuli. This response is captured by the inferred transition rates, which depend on the chosen set of decision variables.

Initialization. To initialize the simulation we used a short stretch of the data right after treatment (post-phase). If time when the empirical observation started is denoted by $t = 0$ (when two of the ants exposed to a pathogenic or non-pathogenic perturbation were placed back to a dish) we initialized the system at time $t_0 = \max_i \Delta_i$ where Δ_i are memory parameters of all decision variables that are part of the model (i.e., for the PLa model we take a maximum of Δ_{P} and Δ_{La}). The initial behavioral states of the ants in the dish are denoted by $\mathbf{x}(t_0)$ and the values of the decision variables are $\mathbf{y}(t_0)$.

Simulation step. We used a standard SSA algorithm, modified to account for the complexity of our model, to simulate the system, following the transitions one by one, as outlined in [1]. To do that, we computed instantaneous transition rates $\lambda(\mathbf{x}(t) \to \mathbf{x}', \mathbf{y}(t))$ (t – current time) and generated two random numbers $r_1 \sim \mathrm{Exp}(1)$ and $r_2 \sim \mathrm{U}([0, 1])$. The first random number set the time at which the next transition occurred using the fact that the stochastic transitions follow a non-homogeneous Poisson process with a rate $\lambda(\mathbf{x}(t) \to \mathbf{x}', \mathbf{y}(t))$. This rate changes in time as the decision variables change. The second random number was used for an identification of an ant, which was about to change its state and identification of the new state. Once the time and type of the next transition was randomly sampled the next simulation step started. Note that the decision variables were recomputed at every time frame since they depend on the past behavior at a fixed-size shifting time window. Thus the process does not possess a Markov property.

Structure of simulations. To obtain a comparable set of simulations to the empirical data we took every experimental replicate and used its initial segment to start a simulation and ran it for the time segment equivalent to the 90 min of the post-phase. This way we obtained a complete set of computer-generated stochastic simulations with the same structure as the experimental data. The experimental data contain enough replicates for each treatment and thus a single complete computer-generated set mimicking the experiment is sufficient for a systematic analysis. However, to understand the accuracy of different models we repeated each stochastic simulations five times.

Supplementary Note 2: Stochastic simulations results

Model selection

Maximum likelihood inference selected the RL models (RLa or RLas) as the best performing models in terms of log-likelihood. But the key question is how do the models compare to the empirical data in terms of forward stochastic simulations of the full probabilistic model of spore removal. Performance of the stochastic simulations is measured by comparing the time traces of the average activity of different groups of ants: ants with the high (F) or low (f) fungal load, control-treated ants (C) and untreated nestmates (N). We also considered three types of activity: performed allo-, received allo-, and selfgrooming. A good model should show similar temporal patterns in all 12 of these time traces. The activity was averaged in 3 min long time intervals in order to eliminate randomness of the temporal data.

Suppl. Fig. 14 summarizes error of the inference method. We use two measures of error:

- L^2 **error of activity traces:** Sum of squared differences between the experimental and simulated traces form the square of this error. The error takes into account the average performed allogrooming, received allogrooming, and selfgrooming of ants sorted by treatment (F, f, C, N) of the stochastic simulation of the model versus experiments for all models. The transition rates for the treated ants were identical to the ones for nestmates, except the $X \to A$ transition, which was reduced by a factor ρ. The parameter ρ was part of the inference and its optimal value is reported in Suppl. Fig. 13.

- L^2 **error on load decay:** Sum of squared differences between the experimental and simulated perturbation load of all the F and f ants at each time form the square of this error. The time traces of perturbation load are computed based on the performed and received activity (either from experimental or simulated data) using the Type II FRM of perturbation load removal.

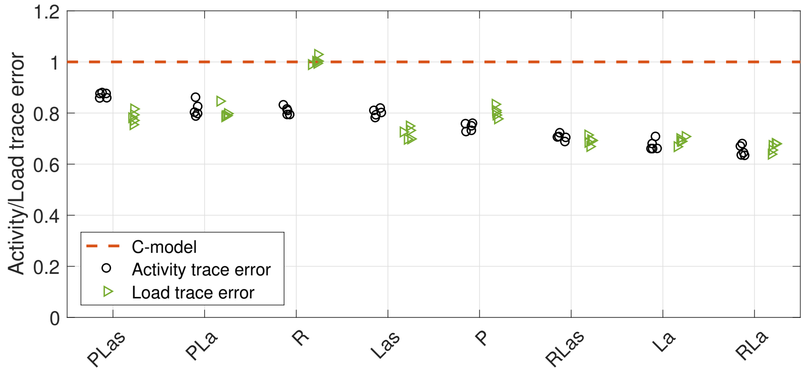

Supplementary Figure 14: Relative activity trace (gray) and relative load trace (green) errors for different models with optimal ρ (ordered by decreasing mean activity error). We performed five simulations for each experimental dish, replicating the whole experimental data set using stochastic simulations five times. For each simulation replicate we took the traces of the performed allogrooming, received allogrooming and selfgrooming, each considered separately for ants F, f, C, and N. The data consisted of 12 activity traces (as explained above), each containing 30 data points (time intervals of 3 min used for averaging). The activity error was computed as an L^2 error between the activity traces in the simulation and experiment. We also computed the L^2 errors in the back-computed perturbation load traces. The back-computing used the Type II FRM and the information about all grooming events. Both panels are scaled with respect to the constant model with the sequential sampling rule. The results are based on all 99 replicates in Suppl. Tab. 1.

Both these measures indicate a good performance of RL models (RLa or RLas), singled out by the log-likelihood performance measure, although the L^2 error (of both types) shown in Suppl. Fig. 14 indicates better performance of models with variable La compared to Las. Since the model comparison based on the full stochastic simulations already integrates the inferred optimal rates (obtained by the log-likelihood maximization) we consider the comparison in Suppl. Fig. 14 more informative than the marginal optimization of the rates in Suppl. Fig. 13. For simplification, as we have now chosen La over Las due to its better performance we from now on refer to the preferred RLa model as our chosen RL model. Note, however, that the model RLas has a very similar performance.

Despite the fact that transition rates in the best performing probabilistic model do not explicitly depend on selfgrooming (they depend on received allogrooming and load seen during allogrooming), the probabilistic model, and consequently also the full stochastic forward model does depend on selfgrooming through two channels. First, the transition model contains the selfgrooming state, which is an integral part of the model,

impacting its dynamics. Second, the variable y_{La} depends on loads of all other recently allogroomed ants. These loads decay during allo- as well as during selfgrooming. Although the coupling between the selfgrooming and the dynamics is not governed directly through the dependencies of the transition rates, indirect effects are present in the model.

Suppl. Fig. 15 shows individual traces of the perturbation load under different model assumptions compared to the inferred perturbation load in the experiments (back-computed from the Type II FRM with grooming events), mirrored for better clarity. These individual load traces are used to compute the load trace error, depicted in Suppl. Fig. 14. We provide two additional sets of statistics for each panel in Suppl. Fig. 15: (a) the mean slope of the linear fit for the simulation-based perturbation load as a function of the experimental perturbation load; (b) the mean R-squared value for these fits, where the mean is taken through all pathogen-exposed ants.

The outcome of the most successful RL model is shown also in Suppl. Fig. 17 (panel B), where we plot both the activity and load traces in time. All plotted datapoints in the activity panel (split by the treatment and activity type) enter the calculation of the activity trace error, depicted in Suppl. Fig. 14. Although for the shortness of the presentation we did not include similar figures for all suboptimal models, we remark that only the models containing load-based dependence variable were able to capture long-term dynamics in the activity traces whereas all other models showed a stationary behavior.

Grooming preference

We simulated the grooming preference rules URAND, SEQ, and MAX for the best performing model RL. In the URAND rule the target for allogrooming was picked at random with equal probabilities, which do not depend on the load of the ants. In this model a contaminated ant has equal probability to be groomed than a nestmate. This serves as a null model for comparison with other models where grooming depends on the pathogen exposure. In the MAX rule the non-focal ant with the maximal load is picked for grooming, provided a nonfocal ant with a detectable load, i.e., larger than the load detection threshold L_0 (see Suppl. Tab. 5), exists. If no non-focal ant exceeds this threshold, the uniform random rule applies. The SEQ model picks an ant by the following simple sequential algorithm: an ant is selected at random and based on its load a decision is made to either accept it as the next allogrooming target or keep probing more ants. If the ant is not accepted, the process repeats. We do not include an explicit waiting time for this exploration but rather assume that its duration is already accounted for in the transition rates. We define the acceptance probability of the ant with load L as a saturation curve

$$p(\ell) = 1 - \exp(-\varepsilon - \ell/L_0)\,. \qquad \text{(Eq.6)}$$

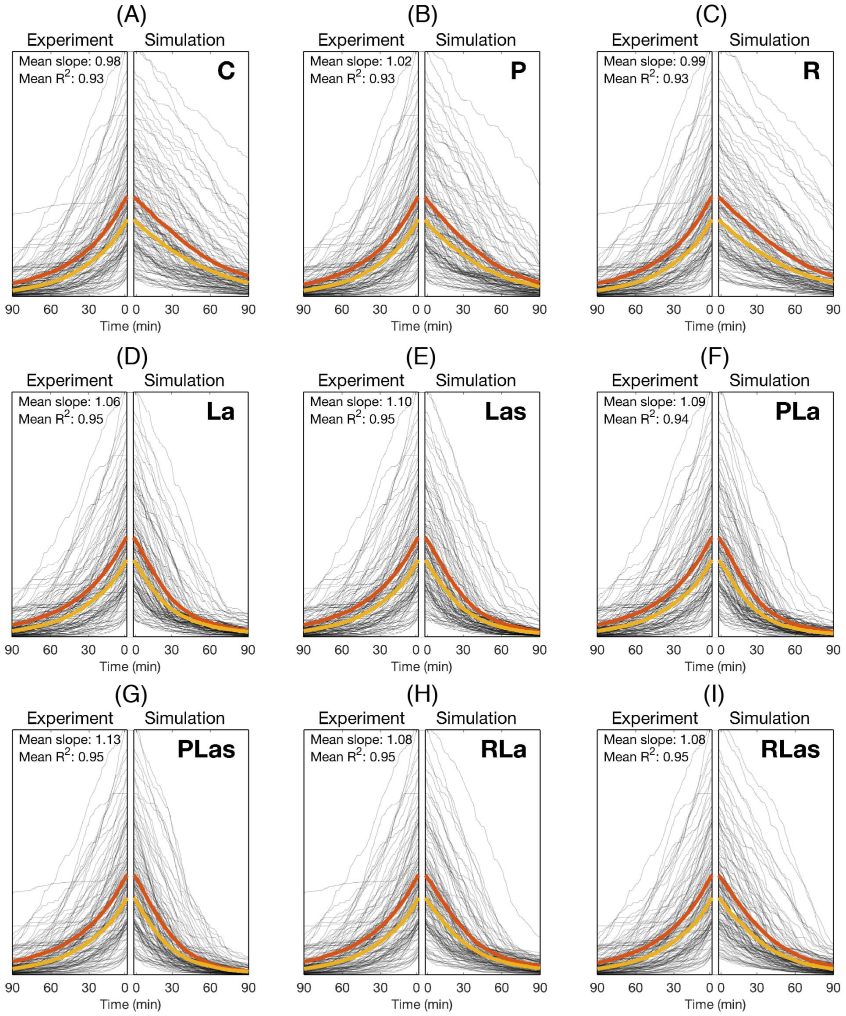

Supplementary Figure 15: Perturbation load in time for the experiment/simulation for all inferred models: the constant model in A, single variable models P, R, La, and Las in B-E, and two-variable models PLa, PLas, RLa, and RLas in F-I, respectively. The load trace of each F and f ant, computed from the initial perturbation loads and the states time (computed using the Type II FRM) is shown in black. The time axis for the experimental traces is mirrored for better visibility. Average of all F/f ant loads is shown in red/yellow. The results are based on all 99 replicates in Suppl. Tab. 1.

This formula has two positive parameters ε and L_0. Empirical evidence shows that even nestmates, which were not perturbed by a pathogenic or non-pathogenic solution, are allogroomed by other ants. The parameter ε captures this load-independent contribution to the probability of grooming an ant. When ℓ/L_0 is very small (e.g., for ants with a small initial load, or after being groomed enough) the relationship (Eq.6) assigns almost

equal probability to each ant to be accepted as an allogrooming target, thus recovering the URAND rule. If ε is small this probability is approximately $1 - \exp(-\varepsilon) \approx \varepsilon$.

On the other hand, the parameter L_0 has a similar interpretation to the corresponding parameter in the MAX model – it determines whether (or how likely) the load of the non-focal ant can be detected by the focal ant – therefore we keep the same notation. The SEQ model combines features of both the URAND and MAX rule. In the regime of $\ell \ll L_0$ the acceptance probability is almost constant (approximately equal to ε) and the rule is analogous to the URAND rule. In the other extreme when $\ell \gg L_0$ the model is similar to the MAX rule. The magnitude of L_0 (relative to ℓ and ε) controls the weight of the load-based relative to the random decision in the SEQ model.

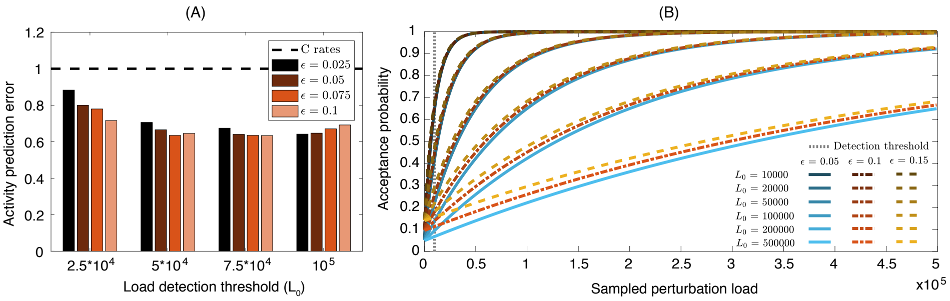

Supplementary Figure 16: (A) Comparison of the activity prediction error in the RL model for the sequential rule with different choices of parameters L_0 and ε. We plot the relative performance to the constant rate model. The results are based on all 99 replicates in Suppl. Tab. 1. (B) Load-dependent acceptance probability for different choices of parameters L_0 and ε.

Suppl. Fig. 16 compares the L^2 error of the activity traces for the sequential rule for a range of L_0 and ε values, indicating the optimal parameter values $L_0 = 5 \times 10^4$ and $\varepsilon = 0.075$. We also plotted the acceptance probability as a function of the parameters L_0 and ε.

Suppl. Fig. 17 shows the activity and the perturbation load traces for the optimal model RL with the optimal preference rule (SEQ), complemented by the same model with the other rules (URAND and MAX). The results show that the speed of removal depends on the grooming preference rule. The URAND rule removes the load at the slowest pace, while the MAX rule removes the load at the fastest pace. The SEQ rule has a removal rate between the extremes of the URAND and the MAX rule. The quantitative ranking between the models in terms of the speed of load removal is an important feature of the proposed models, which is simple to prove rigorously. Moreover, it shows that the rule, which removes the load the fastest is not optimal for the ant colony. We further study the reasons for this suboptimality by including the cost of exploration associated to the probing of the non-focal ants.

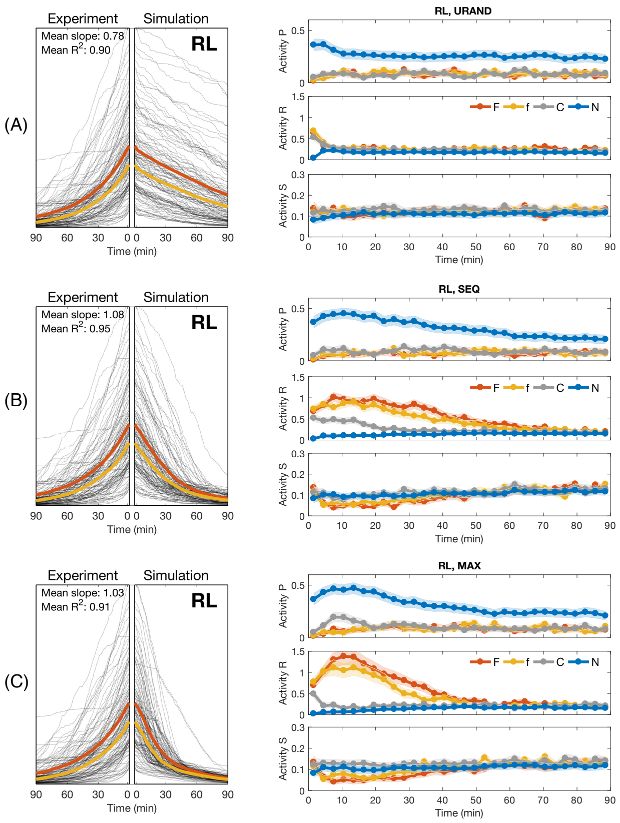

Supplementary Figure 17: Comparison of inferred loads (left) and activity traces (right) in the simulation/experiment for different grooming preference rules for the RL model with an optimal value of ρ. The used grooming preference rules: (A) URAND – uniform random sampling, which removes the load slower than reality, (B) SEQ – sequential load based, which performs similarly to experiments, and (C) MAX – maximum load sampling, which removes the load faster than reality. The panels on the right contain the mean activity traces (filled circles). Shaded areas show the sem centered around the means, which are magnified for better visibility in the plot by a magnification factor of $1.5\times$ for the treated (F, $n = 66$; f, $n = 65$ and C, $n = 67$) and $3\times$ for the nestmate (N, $n = 396$) ants. The activity traces for the performed grooming are constructed as the proportion of time the ant has been grooming others within the relevant time interval of length 3 min. The activity traces for the received grooming are constructed as the proportion of time the ant has been receiving grooming from each ant within the relevant time interval of length 3 min, summed for all other ants (can be larger than 1 if the ant is groomed by more ants simultaneously). The results are based on all 99 replicates in Suppl. Tab. 1.

Null models

We present two variations of the optimal RL model that serve as benchmark models. These two models differ from the optimal RL model only in the decision rule that specifies the groomed ant:

- Sequential probing without load updating. This rule is analogous to the sequential rule; however, the underlying perceived loads of the spore-treated ants (based on which one ant is selected for grooming) are assumed to be constant. Thus, the ants decide who to groom based on initial loads instead of loads updated continually using the Type II FRM. Initial loads are back-computed using Type II FRM (as in the optimal RL model), capturing heterogeneity in the load of ants of the same treatment. This rule is not affected by the performed and received allogrooming, during which ants remove spores from others.

- Model with optimal grooming probabilities. This model assigns a treatment-specific probability to each ant ($p_F + p_f + p_C + p_N = 1$), which is used to decide which of the other ants to groom. The selection of the target ant is proportional to the treatment-specific probability, where the constant of proportionality ensures that the probabilities of all non-self ants adds up to 1. The optimal probabilities were obtained by a random search in the set (p_F, p_f, p_C, p_N) of admissible probabilities, which reached the smallest activity trace error (we ran a single simulation for each experimental replicate in every step of the optimization and used these data to compute the error). The optimal probabilities are $(0.28, 0.32, 0.30, 0.10)$. Note that we did not enforce any relative constraints between the treatment-specific probabilities. The outcome suggests best performance when all perturbed ants are groomed roughly equally likely, while the nestmates being groomed rarely.

Both of the above models perform worse than the optimal model; namely, the error of activity traces is 1.32 times larger in case of the sequential model without updating, and 1.17 times larger in case of the model with optimal grooming probabilities.

Model performance depends on initial spore load variation

Despite the fact that we applied the same volume (0.3 µl) of the same spore concentration to each ant per treatment (1×10^9 spores/ml to each F ant, and 5×10^8 spores/ml to each f ant; see above Pathogen exposure section for details), our ddPCR quantification of $n = 30$ workers per spore treatment to quantify applied spore load revealed that this application procedure includes a variation (sem) of $\pm 8.6\%$ for the F ants and of $\pm 7.0\%$ for the f ants. Hence, even if F ants on average received approximately twice the spores than f ants, this variation means that – depending on chance – the ants paired in some

replicates may have deviated substantially from this ratio. As the relative spore load of two contaminated ants is crucial for understanding the ants' behavioral dynamics, we assessed the impact of this initial spore load variation for our model performance by a set of simulations either taking the variation into account (by initializing the perturbation load of the contaminated individuals in our model with the back-calculated initial spore loads computed per individual per replicate) or neglecting this variation (by initializing our model with the fixed respective medians for initial spore load contamination levels, retrieved from the body samples of the ants directly after exposure (162,000 for the F workers and 89,900 for the f workers)). In both cases, we obtained perturbation loads at later times by dynamically updating the loads using the Type II FRM with fitted parameters according to the activity of the ants in each dish. In Suppl. Fig. 18 we show the results of these simulations. We note that when fixing the values to the initial values, the agreement between the model and the data is much worse compared to the simulations where back-calculated initial loads were used (except for the URAND rule, which performs approximately the same as the analogous model including initial perturbation load variation). This can be easily compared in terms of the relative L^2 activity error. For the optimal RL model including initial load variation the errors reached values 1.08, 0.65, and 0.86 for the preference rules URAND, SEQ, and MAX, respectively (results in Suppl. Fig. 18). On the other hand, keeping the initial loads fixed within the ants' treatment groups led to errors 1.07, 0.80, and 1.18 (results in Suppl. Fig. 18). The L^2 errors, computed relative to the constant model with the SEQ rule, show that initial load variation is an important component in the dynamics of sanitary grooming of the ants.

Minimal model

When inferring the probabilistic model from data it is essential to keep in mind the tradeoff in the resolution of the transition rates. On one hand, choosing a fine binning of the rates may potentially inform us about the actual shape of the rate functions in terms of the decision variables. On the other hand, with limited volume of the dataset detailed resolution of the rates becomes prohibitive and leads to overfitting, i.e., the inferred model may work well on the provided dataset but loses its prediction capability.

To obtain a good resolution of the transition rates while avoiding overfitting we picked three categories for each decision variable. This choice leads to $9 \times 8 = 72$ parameters of the transition model (with one additional parameter ρ), which may not all be necessary. To show robustness of the optimal RL model we explored a reduced model with a less detailed resolution on the key transition rates $X \rightleftharpoons S$ and $X \rightleftharpoons A$ and constant rates, where less data are available. Suppl. Fig. 19 shows the results for this model with 29 parameters including ρ. We have also performed a comparison with the minimal PL model, analogous in the number and structure of parameters to the minimal RL model. Interestingly, lower resolution of transition rates significantly worsens the outcome of the PL model compared to the optimal RL model (3.16 times larger L^2 error

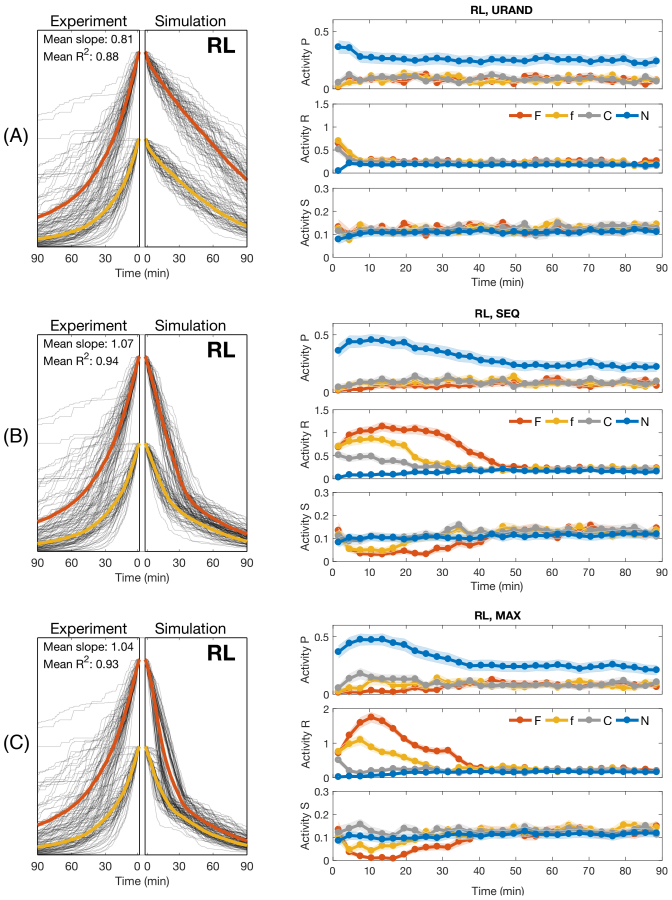

Supplementary Figure 18: Comparison of inferred loads (left) and activity traces (right) in the simulation/experiment for different grooming preference rules for the RL model with an optimal value of ρ. Here instead of computing the initial load from the Type II FRM using the knowledge of the loads at the end of the experiment and the activity throughout we initialized the loads using the experimentally measured median values of the spore load on the bodies of the F and f ants. The used grooming preference rules: (A) URAND – uniform random sampling, which removes the load slower than reality, (B) SEQ – sequential load based, which performs similarly to experiments, and (C) MAX – maximum load sampling, which removes the load faster than reality. The panels on the right contain the mean activity traces (filled circles). Shaded areas show the sem centered around the means, which are magnified for better visibility in the plot by a magnification factor of 1.5$\times$ for the treated (F, $n = 66$; f, $n = 65$ and C, $n = 67$) and 3$\times$ for the nestmate (N, $n = 396$) ants. The results are based on all 99 replicates in Suppl. Tab. 1.

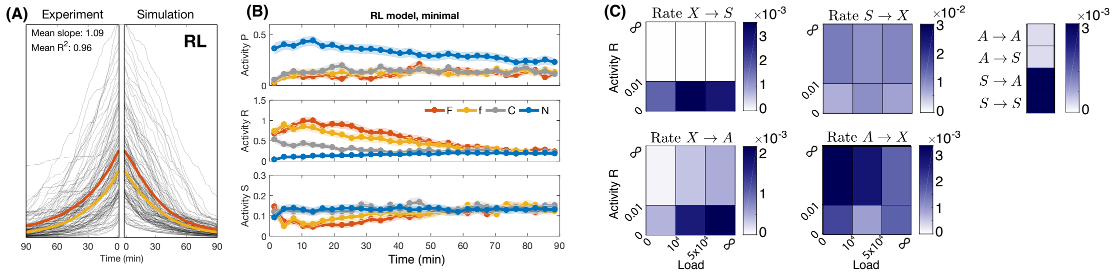

Supplementary Figure 19: The minimal model. Results of the RL model with reduced number of inferred parameters in the transition rates from 73 (eight transition rates, each resolved to nine bins with nine coefficients, and $\rho_{\text{opt}} = 2.38$) to 29 parameters, as shown in panel (C). Performance of the RL model (A-B) in terms of all measures considered (activity trace, load trace error) is comparable to the model before reduction. (B) contains the mean activity traces (filled circles). Shaded areas show the sem centered around the means, which are magnified for better visibility in the plot by a magnification factor of 1.5× for the treated (F, $n = 66$; f, $n = 65$ and C, $n = 67$) and 3× for the nestmate (N, $n = 396$) ants. The results are based on all 99 replicates in Suppl. Tab. 1.

in the activity traces) while keeping an almost unchanged performance of the RL model (1.1 times larger L^2 error in the activity traces). This suggests a more robust RL model compared to the PL model.

Exploration-exploitation tradeoff

Why is collective spore removal behavior of the ants better explained by the SEQ model rather than the MAX model, which removes the spore load faster? Here we present a simplified model of the spore removal behavior that introduces an exploration cost as a realistic feature to explain the apparent suboptimality of the spore removal mechanism. This simple added feature allows us to understand the ant's behavior not as suboptimal but rather as optimizing a different cost. Moreover, the proposed simple model allows us to extend the analysis to more than six ants in a single dish and study the scalability of different grooming decisions to larger groups of ants.

Exploration is needed for the focal ant to learn how much perturbation load the nonfocal ants carry and to make an informed decision. However, exploration leads to both benefits and costs. The benefits, measured by the total amount of perturbation load removed by allogrooming, are larger when the ants with high loads are groomed. On the other hand, the cost is directly proportional to the number of ants that are probed before the grooming decision is made.

Under which conditions do the net benefits of exploring exceed the cost of time delay before the grooming begins? To study this question we formulate simple models for two

grooming strategies: one, where the focal ant needs to acquire complete information about all other ants and consequently grooms the ant with the highest perturbation load (analogous to the MAX rule), and the other where the focal ant decides probabilistically whether to accept a randomly selected ant for grooming based on its perturbation load (analogous to the SEQ rule).

Naturally, the optimality of one strategy or the other depends on the specifics of the model. We aim to keep maximal simplicity of the models but at the same time to capture the information allocation realistically. We assume that

1. The interacting group contains $N \geq 6$ ants of which $N_L = N/3$ have a nonzero initial load with value ℓ^*. The case $N = 6$ corresponds to the experimental data studied in this work. We picked N to be divisible by 3.

2. We incorporate Type II FRM kinetics for the load decay during allogrooming as in (Eq.3) with parameters $v = 1$ and $K = 100$.

3. Each ant is either grooming or exploring at all times (no resting).

4. Exploring a single ant (i.e., learning its current load) takes a fixed amount of time t_E. During this time the exploring ant cannot groom (exploring and grooming are exclusive states).

5. Duration of uninterrupted grooming t_G is fixed.

6. Grooming or exploring can occur at the same time as receiving grooming from others. They are not considered exclusive events.

Maximal rule. In the maximal rule each ant explores all other ants one after another and subsequently grooms the nonfocal ant with the highest load. Since at all times there are at least two ants with nonzero load ($N_L \geq 2$) each ant is grooming productively (the recipient has a nonzero load) at all times. Thus, the fraction of productive grooming time is

$$\frac{t_G}{Nt_E + t_G} = \frac{1}{1 + N\tau} \qquad \text{(Eq.7)}$$

for $\tau = t_E/t_G$.

Sequential rule. In the sequential rule each ant repeats the behavioral sequence: probe to learn the load ℓ of the nonfocal ant, evaluate the acceptance probability $p(\ell) = 1 - \exp(-\ell/L_0 - \varepsilon)$ (Eq.6) and decide probabilistically whether to groom or move to another random ant to restart probing. Note that the higher the load ℓ the closer the acceptance probability is to 1. Also, even the ants with a zero load have a small chance $p(0) \approx \varepsilon$ of being accepted. When $\ell/L_0 \gg \varepsilon$ (regime of large initial load) then the total amount of

Supplementary Table 7: Parameters in the exploration-exploitation model

Notation	Value	General parameters
N	6-150	Number of ants in the interacting group
N_L	$N/3$	Number of ants with a nonzero initial load at time $t = 0$
ℓ^*	100	Nonzero initial load of N_L pathogen contaminated ants
t_E	1, 10, 100	Time needed to probe one ant and obtain information about its current load
t_G	1000	Duration of uninterrupted grooming
v	1	Decay rate in the Type II FRM
K	100	Load at which the Type II FRM decay is half-maximal
		**MAX strategy parameters**
M	N	Number of probed ants
		**SEQ strategy parameters**
L_0	1	Reference load in the SEQ rule
ε	0.01	Parameter of the acceptance probability in the SEQ rule

time spent productively grooming is approximately

$$\frac{p(\ell)\frac{N_L}{N}t_G}{t_E + p(\ell)\frac{N_L}{N}t_G + (1 - p(\ell))\left(1 - \frac{N_L}{N}\right)t_G} \approx$$
$$\approx \frac{1}{1 + 3\tau + 2\exp(-\ell/L_0 - \varepsilon)} \qquad \text{(Eq.8)}$$

A simple inequality between expressions (Eq.7) and (Eq.8) involving parameters N, τ and ε determines whether the MAX or SEQ strategy works better at removing the load from the group of interacting ants.

- If $(N - 3)\tau > 2\exp(-\ell/L_0 - \varepsilon)$ then the SEQ strategy removes load faster then the MAX strategy, otherwise the MAX strategy is more efficient at removing spore load.

The first consequence of this result is that given all other parameters fixed (t_E, t_G, ε, ℓ/L_0) as the number of ants grows the MAX rule gets relatively less efficient compared to the SEQ rule. This is because obtaining complete information about the whole community becomes more expensive as N grows. The relative performance of the MAX strategy for systems of a given size N is better when τ is smaller, which means that the exploration time is relatively small with respect to the grooming time. As expected, the initial load modulates the threshold between the net benefits of the two rules with very large relative initial load ℓ/L_0 leading to a better performance of the SEQ rule as compared to the MAX rule. Of course, our calculation relies on a couple of assumptions (large nonzero loads, small ε, etc.), which may be violated later in the dynamics.

Next we explored a class of decision functions to commit to grooming $p(\ell)$, to identify which features of these functions matter when there is an exploration cost and which ones lead to efficient colony-wide choice. We tested the following classes of functions:

A0: $p(\ell) = p_0$ for $p_0 = \{0.1, 0.2, 0.3, \ldots, 1\}$;

A1: $p(\ell) = p_0 + (1 - p_0) * \frac{\ell}{100}$ for $p_0 = 0.1, 0.9$;

A2: $p(\ell) = p_0 + (1 - p_0) * \frac{\ell}{100}$ for $p_0 = 0.1, 0.9$ with $p(0) = 0$;

A3: $p(\ell) = p_0 + (1 - 2p_0) * \frac{\ell}{100}$ for $p_0 = \{0.1, 0.2, 0.3, 0.4, 0.5\}$ with $p(0) = 0$.

Along with the function $p(\ell)$, we also varied the parameters t_E and t_G to reflect the importance of the exploration cost. Randomness was controlled by the total number of ants (third of which were infected) and by the amount of initial load ℓ^*, which was chosen to be uniformly randomly distributed in interval $[0, 100]$. The performance of the simulations was measured by the rate of load reduction in the group of ants at a given time (same time for all simulations in direct comparison). The performance was computed based on sufficiently many replicates (up to 1000).

We found that

1. When the function $p(\ell)$ is constant (A0), its increased value leads to greater removal of spores.

2. The property described above is not valid generally. The comparison between the functions of A1 shows more spore removal when the function $p(\ell)$ is lower (while steeper).

3. The effect of $p(0)$ is very important since $p(0)$ being small or even equal to zero guarantees that time is not wasted for grooming of nestmates. The comparison in A2 with enforced constraint $p(0) = 0$ recovers an increasing property, i.e., higher values (flat function) lead to more spore removal.

4. Stochasticity is key to determining the optimal steepness of the function. The comparisons in A3 show that large stochasticity (N is small) favors a less steep function, while on the contrary, low stochasticity (N large) favors a steeper function.

5. The high exploration cost, in general, leads to slower spore removal.

6. The preferred shape of $p(\ell)$ depends on the time horizon of the simulation. This is because for steep functions the ants remove most of the load fast while having difficulty removing the rest of the load, whereas less steep functions remove the spores at approximately the same rate, independent of the load.

Fig. 20 compares two simulations for functions $p(\ell)$, differing in their slope. In the simulation corresponding to the steeper slope, the ants with the highest load are groomed more initially, reducing the high loads significantly. Consequently the frequency of grooming declines leading to a slower spore removal. In the simulation with the shallow function the rate of grooming stays approximately the same until the ants run out of load completely.

Supplementary Note 3: Statistical data analysis

Individual and social sanitary behaviors

For each of 594 ants in 99 replicates (Suppl. Tab. 1), we analyzed the 'effective time' in which it performed each sanitary behavior, i.e. the proportion of time allocated to selfgrooming its own body, resp. head, uptake of its own poison, and allogrooming others, for any given period. We also determined the effective time in which each ant received allogrooming from its group members, but here took into account that one ant could be groomed simultaneously by several ants, and calculated the sum of the time allocated by different ants to the receiver for any given period (in which case the obtained value may exceed 1). We tested if the effective time allocated to each behavior differed between the pre- and the post-treatment period by use of paired Wilcoxon tests according to individual treatment, i.e. high-load (F), low-load (f), control-treated (C) and untreated (N). To avoid pseudoreplication we obtained a single value per individual treatment and replicate, by averaging the values of all ants in the same group which had received the same individual treatment (i.e. one average value for the two F-individuals in FF, the two f-individuals in ff, the two C-individuals in CC, and the four N in any group). As we observed multiple behaviors for each individual (selfgrooming body and head, poison uptake, allogrooming performed and received), we accounted for multiple testing. We report the adjusted p-values, V values and effect sizes in Suppl. Tab. 2 and display the change in body selfgrooming, performed and received allogrooming (in Fig. 1), and of poison uptake behavior (in Suppl. Fig. 2) as the difference between effective time in the post- and pre-period.

To identify if nestmate selfgrooming in the post-period depended on preceding allogrooming events, we determined the fraction of time that the nestmates spent on selfgrooming their head and body following either performed or received allogrooming in a time window of max. 3 min or until the next allogrooming was performed resp. received. This was separately obtained depending on treatment of the partner ant (spore-treated F or f, control-treated C, or other nestmate N). For the control-treated individuals, we also separate the C individuals that were paired with a spore-treated (F or f), i.e. were in a group that contained spores, or was paired with a second control-treated individual in a pathogen-free group treatment. We observed a total of 13253 events of

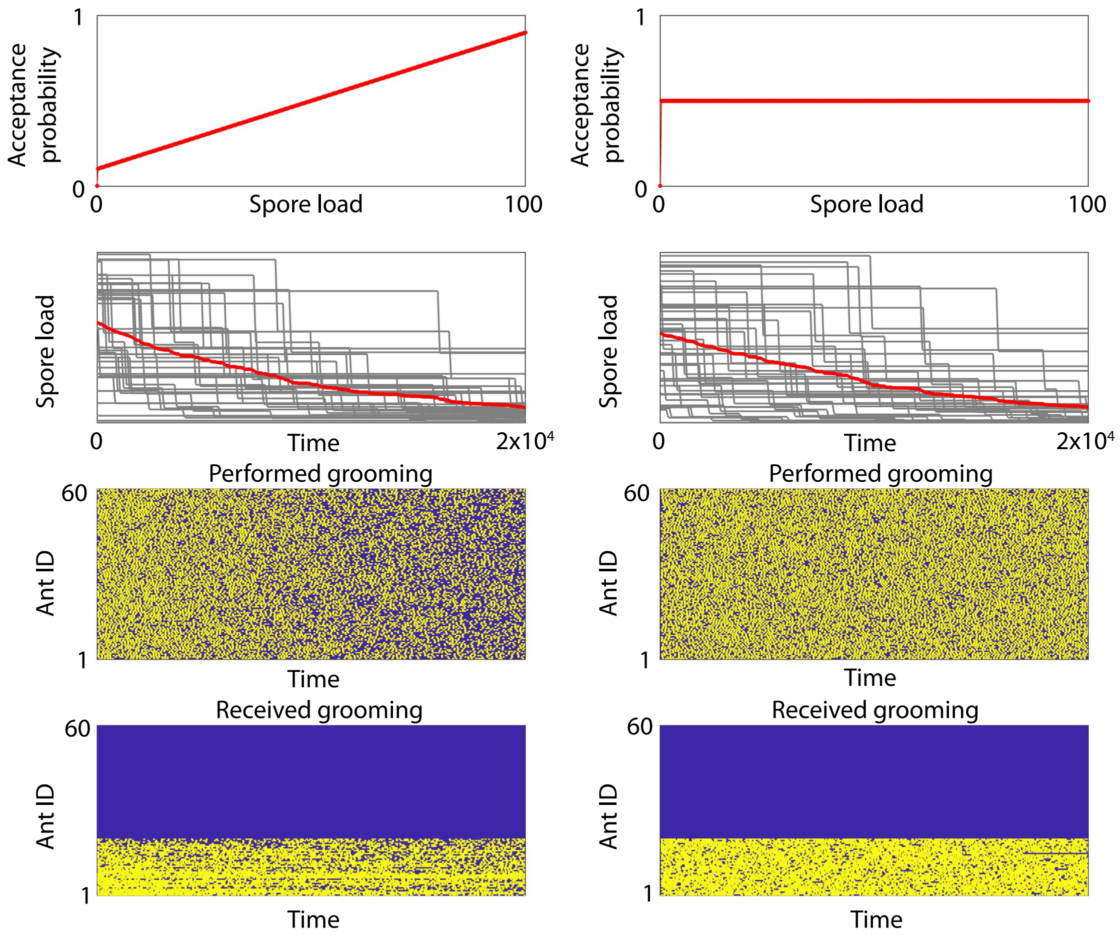

Supplementary Figure 20: Example simulations of the exploration-exploitation model for acceptance probability $p(\ell)$ from class A3. From top to bottom: acceptance probability, time-resolved load paths (gray) with their mean (red), raster plots of the performed and received grooming (grooming – yellow, idle state – blue). (Left) $p_0 = 0.1$, (Right) $p_0 = 0.5$.

N performing grooming and 4934 events of N receiving grooming. We performed a Kruskal-Wallis test each for performed and received grooming. For the significant case of performed grooming we then carried out all-pairwise Dunn posthoc comparisons and adjusted the p-values for multiple testing (Suppl. Fig. 3).

We further analyzed whether there is a relationship between the grooming that an ant received by others recently, and its own propensity to groom others in the next moment. To this end, we calculated the log odds ratio to perform allogrooming oneself as a function of time since the last received grooming by others (time lag). The log odds ratio was defined as a ratio of the probability that the performed grooming follows after received grooming to the probability that the performed grooming follows after non-receiving, as a function of the elapsed time (data binned into 5 sec bins; see Eq. 10). We first determined this effect of social feedback between grooming received in the recent past and performed in the next moment in the absence of any treatment for all ants in the pre-treatment phase ($n = 594$ ants from 99 replicates). To determine the effect of the pathogen, we considered all 82 replicates, in the post-treatment phase, from the treatment groups with at least one spore-treated individual (Suppl. Tab. 1), separately for the 164 treated individuals and their untreated nestmates ($n = 328$ N; Fig. 6b). We also analyzed the treated individuals separated by their treatment (Suppl. Fig. 9; 66 F-individuals, 65 f-individuals, 33 C-individuals from the 82 replicates). The dependency of grooming preference on time lag was assessed by Spearman-rank correlations, as detailed below.

Log-odds ratio to groom as a function of time since last received grooming

The spore removal ant behavior is well captured by the probabilistic models, which involve two decision variables: the last nonzero load seen during allogrooming (within previous Δ_{L} seconds) and the proportion of time the ant was allogroomed (within previous Δ_{R} seconds). Here we look at how previous received allogrooming events impact further decisions to groom the others. We do that by calculating the log-odds ratio to groom, comparing the probability of grooming others at time t given last received grooming Δt ago with the probability of grooming others at time t given not receiving Δt ago and after, all as a function of Δt.

To be specific, we bin the activity data of all ants in all the dishes for each treatment (both for the experiments and simulations) into short windows (5 seconds). We are interested in

(a) The times when the ant receives allogrooming from others (received allogrooming event is denoted by E_R).

(b) The times when the ant starts allogrooming others (performed allogrooming event

is denoted by E_P).

For each window we capture information of whether at least one E_P event occurred in this window, and similarly, whether at least one E_R event occurred in this window. For convenience we denote by $E_{P'}$ and $E_{R'}$ complement events to events E_P and E_R, respectively. We do not distinguish between multiple events of the same type within a window and we do not use the information of which of the E_P, E_R events within one window came first if both occurred. Next we define for each $\Delta t = 1, 2, \ldots$

- $N_{RP}(\Delta t)$ – the number of times the E_P event occurred exactly Δt windows after the last E_R event.
- $N_{RP'}(\Delta t)$ – the number of times the $E_{P'}$ event occurred exactly Δt windows after the last E_R event.
- $N_{R'P}(\Delta t)$ – the number of times the E_P event occurred exactly Δt windows after the $E_{R'}$ event.
- $N_{R'P'}(\Delta t)$ – the number of times the $E_{P'}$ event occurred exactly Δt windows after the $E_{R'}$ event.

Note that the above integers account only for those events where between the selected event types there is no other E_R event, i.e., we condition on the last E_R event. The E_P events do not intercept the considered train since the last E_R event still plays an important role for the decision of when and whether to groom even if E_P events were performed in the meantime.

The log-odds ratio to groom as a function of the time since last receiving grooming from others is then computed as

$$LOR_{\Delta t} = \log \left[\frac{N_{RP}(\Delta t)}{N_{R'P}(\Delta t)} \times \frac{N_{R'P}(\Delta t) + N_{R'P'}(\Delta t)}{N_{RP}(\Delta t) + N_{RP'}(\Delta t)} \right] \qquad \text{(Eq.9)}$$

Suppl. Fig. 10 shows the $LOR_{\Delta t}$ computed separately for all types of treated ants: the ones treated with a large spore-content pathogenic suspension (F ants), low spore-content pathogenic suspension (f ants), and the ants treated with the non-pathogenic solution (C ants). The error bars were obtained by repeatedly withholding four replicate dishes from each treatment (24 dishes withheld, 75 remaining, cycling pattern). As Suppl. Fig. 10 shows that the three types of treated ants do not behave differently, all treated ants are combined and shown in comparison to the nestmates and the pre-phase ants in Fig. 6b (of the main text).

Spore-load dependent nestmate grooming preference

For every grooming event in which an untreated nestmate chose to groom one of the two spore-treated ants ($n = 5001$ individual grooming choices performed by the 196 N from the 49 replicates with two spore-treated individuals: in detail 1695 grooming events in FF, 1787 in Ff, 1519 in ff), we computed the current spore loads on both treated ants (at the start of the following 30 second-window), using the approach described above. For each grooming choice we then derived the current 'spore proportion on groomed ant' as the current spore load of the targeted ant over the total current spore load of both treated ants. We compared the observed distribution of these spore proportions to a null expectation, i.e. the distribution of this proportion under the expectation of uniformly random choice (Fig. 3d). To this end, at each time point when a nestmate groomed any of the two treated ants, we simulated random choices ($n = 100$), where the nestmate selects one of the two treated ants uniformly at random. We used a Kolmogorov-Smirnov test to statistically compare the observed and expected distributions (Fig. 2c).

We further analyzed whether the duration of the individual grooming events depended on the current spore load proportion of the groomed ant (Fig. 2d). We binned all grooming events into ten categories of current spore proportion on the groomed ant (0-0.1, 0.1-0.2, $\ldots$, 0.9-1.0; with 1 representing the extreme case where the groomed ant would have had all and the non-targeted ant had zero load) and counted how many events of particular duration (in 10 sec steps) fell into each category. To test for a possible relationship of the duration of grooming events with the spore load proportion on the groomed individual, we performed all-pairwise two-sample Kolmogorov-Smirnov tests between each of the categories resulting in 45 comparisons, which were adjusted for multiple testing. Statistics were performed on all grooming data, while Fig. 2d displays only the 90% of the grooming events with grooming duration shorter than 2 min.

Out of the grooming choices by the nestmates towards the two treated ants, we calculated the log odds ratio to groom the individual with the current higher spore load (total of 8129 grooming decisions by 328 N, out of which 5001 grooming events by the 196 N in the 49 replicates with two spore-treated individuals FF,Ff, ff; and 3128 grooming events by the 132 N in the 33 replicates with one spore- and one control-treated FC,fC). We calculated Spearman-rank correlations to determine how the log odds ratio to groom the currently higher-load individual depended on the spore load difference between the two treated individuals (fairly binned into ten categories for each treatment group, so that each category of spore-load difference contained roughly the same number of grooming events), for all treatment groups (Fig. 6a) and separately for groups containing only one or two spore-treated individuals (Suppl. Fig. 9). We show the observed values in comparison to the theoretical prediction, as calculated from Eq.10, described below.

The optimal grooming rule in the SEQ model is described by the acceptance probability $p(\ell)$ of an ant with a load ℓ of a form (Eq.6). Here we consider an approximation

where the ants have a full knowledge of the loads of all the other ants and they decide at once who to groom based on grooming probabilities for the ants in the dish are proportional to $p(\ell)$. Such grooming probabilities are consistent with the SEQ rule, which is valid in the limited knowledge scenario. Note that the sum of these $p(\ell_i)$ through all ants i in a dish does not need to equal one.

Based on this knowledge we may compute the log-odds ratio (LOR) to groom an ant with a load ℓ as opposed to grooming an ant with a load ℓ_1 in the form

$$LOR_{\Delta\ell} = \log \frac{1 - \exp(-\varepsilon - \ell/L_0)}{1 - \exp(-\varepsilon - \ell_1/L_0)} \quad \text{(Eq.10)}$$

We use this theoretical prediction of the log-odds ratio as a function of $\ell - \ell_1$ to fit the data in Fig. 6a (of the main text) and in the Suppl. Fig. 9 along with the optimal values of the parameters $L_0 = 50,000$ and $\varepsilon = 0.075$. We optimize the only parameter ℓ_1 to find the best fit, obtaining $\ell_1^* = 41638$ (R-squared = 0.26, CI = $[31508, 51768]$) in the case where we fit to all treatment groups, $\ell_1^* = 52479$ (R-squared = 0.34, CI = $[42659, 62300]$) when we fit to treatment groups with two spore-treated ants (FF, Ff, ff), and $\ell_1^* = 33124$ (R-squared = 0.27, CI = $[17148, 49101]$) when we fit to treatment groups with a single spore-treated ant (FC, fC). Interestingly, the $LOR_{\Delta\ell}$ curve saturates at a positive value instead of having an unbounded growth.

We also tested if the nestmate preference to groom the higher-load individual could be predicted by the spore load difference of the two spore-treated individuals based on their applied initial spore load (Suppl. Fig. 7; i.e. if the ants would keep a long-term memory of the initial spore load as compared to basing their decisions on the dynamically changing current spore loads, shown in Fig. 6a, Suppl. Fig. 8). This analysis was restricted to groups in which the two treated ants received different initial spore doses (Ff, FC, fC; $n = 4915$ grooming events by 196 N from 49 replicates), where we calculated the log odds ratio that the nestmates groom the individual with the higher applied spore dose (F in Ff, F in FC, f in fC), separately for the early, mid and late periods of the experiment, each containing a similar number of grooming events (note that most grooming was performed at the beginning of the experiment, leading to unequal time intervals).

Relationship between performed grooming and head-sampled spores

We determined the presence or absence of the GFP- and RFP labelled spores in the head samples of each nestmate ant (N) from the 82 groups with at least one spore-treated individual (FF, Ff, ff, FC, fC; $n = 328$ N, 196 of which had contact to two spore-treated individuals, leading to a total of $n = 524$ data points for label-dependent spore collection and performed grooming) at the end of the experiment. For each nestmate, we also quantified the total time (min) it had groomed the ant treated with the

respectively-labelled spores over the course of the experiment. To test whether the duration of grooming performed towards the ant with the respectively labelled spores (GFP vs RFP) predicted the presence of these spores in the head of nestmates (Suppl. Fig. 4a), we ran a logistic regression (GLMM with binomial error distribution and logit link function) for each of the two labels with the binary variable of presence/absence of spores in the head as the dependent variable and the total time grooming towards the ant, onto which these labeled spores had been applied, as a continuous predictor. To address potential non-independence in our data (i.e. four nestmates per group), we included a random intercept term of ants nested within group in our model.

In addition to the presence/absence of spores, we tested for a quantitative relationship between the number of spores of a given label from the nestmate's head sample and the duration it had groomed the ant of the respective spores, for different time intervals backwards from the end of the experiment (i.e. when the spore load was determined). To this end, we performed Spearman-rank correlations between the number of spores and total time groomed for the full interval of the experiment (0-90 min) and for increasingly shorter periods (each shorter by ten minutes, i.e. each starting ten minutes later and all ending at minute 90). We obtained 9 correlations, which were corrected for multiple testing. This allowed us to determine how the strength of the correlation (Spearman rho) depended on the time interval before spore quantification at the end of the experiment (Suppl. Fig. 4b).

Pellet formation depending on group treatment and experimental period

We determined the number of pellets that each group of six ants produced, and which we collected from the dish at the end of the experiment. We found that no pellets were produced in the groups where both individuals were treated with the control-treatment. For the groups containing at least one pathogen-exposed individual (FF, Ff, ff, FC, ff; $n = 82$ replicates) we tested whether pellet number (including the 4 replicates which did not produce any pellets) depended on group treatment (Suppl. Fig. 5a). We performed simple linear regression of the number of pellets per replicate with treatment as a single predictor, followed by Tukey comparisons. We also tested whether the number of spores per pellet (total number of spores divided by the number of pellets in the pool) differed among treatment groups, using a Kruskal-Wallis test.

We also observed pellet expulsion events by the ants, and noted that ants sometimes take up a recently spit pellet again into the mouth, to expel it again. To test if the time that a nestmate had spent allogrooming between observed consecutive pellet productions depended on the phase of the experiment (i.e. the first, second and third 30 min-period of the post-treatment period; Suppl. Fig. 5b), we therefore excluded pellet expulsion events without preceding grooming activity. Also, we only took replicates into

account where the discrepancy between retrieved pellets and observed expulsion events was smaller than 0.6 (absolute difference between number observed and retrieved, divided by their maximum) and had to exclude 2 expulsions due to faulty frame numbers in our manual annotations. Out of the 143 observed cases where nestmates expelled a pellet, 98 were the first pellets while the remaining 45 being the 2nd ($n = 33$), 3rd ($n = 11$), or 4th ($n = 11$) pellets produced by the same individual. As the filling state of the infrabuccal pocket at the beginning of the experiment could not be controlled for and it may also contain non-pathogenic detritus, we did not analyze the time to first pellet expulsion and only included the later pellets, which were formed completely during our experiment, into the analysis. As this conservative approach led to a large reduction in sample size, we repeated the analysis including the first pellet, which did not qualitatively affect our results (details not shown). We compared grooming time before expulsion among the three time periods with a Kruskal-Wallis test, performed pairwise comparisons using unpaired Wilcoxon tests and corrected for multiple testing.

Relationship between nestmate grooming preference and spore removal efficiency

For each group with at least one spore-treated individual, we determined the number of spores removed by the nestmates by allogrooming (i.e. the sum of spores retrieved from their head samples at the end of the experiment, $n = 328$ N head samples) and by pellet formation in the group ($n = 77$ pellet pools) and calculated its proportion with respect to the total spores quantified from all ants and pellets in the replicate at the end of the experiment. We tested if spore removal correlated to the degree of grooming preference towards the currently higher-load individual in the group, i.e. the ratio of the performed grooming events towards the treated individual with the higher current spore load compared to all grooming events towards both treated ants (from five independent replicate simulations; see below). To this end, we fitted a linear functional model for either all groups with at least one spore-treated individual (Fig. 7a; $n = 82$ replicates) or separately for groups with two spore-treated individuals (FF, Ff, ff; $n = 49$ replicates) or for groups with one spore-treated and one sham-treated individual (FC, fC; $n = 33$ replicates; Suppl. Fig. 11).

The optimal RL model, despite its stochasticity, shows a consistently great agreement with the empirical data of ant behavior. Here we test how well our model agrees with our empirical data of how many spores were removed by each ant group (dish) by allogrooming, i.e. the sum of spores (a) collected by the nestmates in their heads and (b) expelled by the ants in form of pellets. Note that we cannot avoid some errors, as the pellet pool per dish will also contain some spores that were expelled after selfgrooming, yet we expect this to be a small fraction, only. Moreover, we identified that the number of spores quantified from the head samples very accurately reflect the

number of spores collected in the ants' infrabuccal pockets as only minimal amounts were still found on the outside of the head capsule at the end of the experiment (see microscopy results above). Note also that these spore numbers do not contain the spores selfgroomed by the treated ants themselves nor any spores which may have fallen off from the spore-treated ants to the environment [3].

We report the correlation between the following two quantities:

- Grooming preference – for each ant group (dish) we determine the grooming preference performed by the nestmates by looking at the two treated ants and evaluating for all allogrooming events they received by the nestmates at the time of their beginning (the time of making the choice of whom to allogroom) whether the choice was correct or not. The correct choice is defined as grooming the ant with the higher current spore load, back-computed from the experimentally measured terminal load on their bodies using the Type II FRM kinetics (with a value 0 for the C treated ants). The grooming preference is computed as the proportion of the grooming bouts directed towards the individual with the higher current spore load on all grooming bouts performed towards the two treated ants.

- Spores removed by allogrooming – Number of spores removed by allogrooming by the nestmates and that are expelled as pellets, i.e. sum of the spores quantified by PCR from all nestmate head samples and in the pool of all expelled pellets of the dish at the end of the experiment. This absolute number of removed spores is scaled by the initial amount of spores applied to the pathogen-treated ants of the replicate dish (medians (162,000 for the F workers and 89,900 for the f workers), hence representing the relative spore removal efficiency.

For each replicate the above measures result in a single planar dot in Suppl. Fig. 11. Since we use simulations to compute the grooming preference, we can use each of the five performed replicate simulations of the optimal model to quantify the error of this measure. Similarly, the removed spores contain an error, associated to the fact that part of the spores are organized into integer number of pellets. The size of the pellets (on average approximately 1500 spores each) results in a granularity, which introduces a binomial error, plotted for each datapoint in the figure. In summary, the error in both the vertical and the horizontal direction in Suppl. Fig. 11 were incorporated into the linear fit using the linear functional model. We use the approach of [4] to find the parameters of the fitted line using an iterative procedure. The output of the method is the R-squared and the confidence intervals of the fitted parameters and their p-values, the former (if significant), reported in Suppl. Fig. 11.

Spore removal based on presence or absence of choice

We compared the amount of spores removed in our follow-up experiment of a group of ants with free choice vs. groups with no choice, which could not choose between different treated individuals. We consider four types of groups with free choice. Each of them consists of four nestmates (4N) and two treated ants; one of them was always treated with the high dose F, and the second with either (i) the control suspension C (easy choice), (ii) a lower spore dose (1/4 F or 1/2 F; moderate choice difficulty), or (iii) with the same dose F (difficult choice). We compare the amount of spores removed in the 20 min of the experiment in the groups of free choice with the no-choice groups, which consisted of half a group of 4N plus two treated ants, that is, only 2N plus one treated with either of the three spore dosages (F, 1/2 F, or 1/4 F). The spores removed in the 4N-F,F group are compared with the spores removed in two 2N-F experiments. While the number and types of ants are the same, the no-choice experiment runs the two halved groups separately and does not allow the ants to choose between more than one treated ants. We similarly compare the groups 4N-F,1/2 F with combined 2N-F and 2N-1/2 F, the group 4N-F,1/4 F with combined 2N-F and 2N-1/4 F, and 4N-F,C with combined 2N-F and 2N-C.

The test is based on R, computed as the ratio of the spores removed in the free-choice situation to the sum of the spores removed in the two respective no-choice situations, whereby we had quantified the removed spores as all spores in the heads of the nestmates and expelled as pellets (see Methods). We used 14 replicates of each situation (4 choice and 3 no-choice situations) and computed the value of R in all the four above-mentioned pairwise comparisons, as well as for the combined ratio for all treatment combinations computed as the mean of the treatment-specific ratios. We tested our *a priori* expectation that free choice would allow the ants to remove more spores than when choice was prevented, which was based on our findings in the main experiment that better choosing groups remove more spores than worse-choosing groups (Fig. 7a), by computing the one-sided significance of the statistics R using bootstrap given the following algorithm (for simplicity we outline the algorithm for the example of 4N-F,1/2 F versus 2N-F and 2N-1/2 F):

1. Construct a table/column T_1: the spores removed with free choice (14 replicates of 4N-F,1/2 F). Permute their order randomly.

2. Construct a table/column T_2: randomly sample 14 replicates from the 2N-F and 14 replicates from the 2N-1/2 F experiment with repeating. The table contains 28 numbers in total. Permute randomly their order and split to two columns of 14 (randomizes the treatment group). Add the columns to obtain a randomized total amount of spores removed in two experiments with constrained choice T_2 (14 resulting numbers).

3. Switch the top halves of T_1 with T_2 (already randomized, this randomizes the choice/no-choice scenario) and randomize the order in the columns again.

4. Add the elements of each table. Compute the ratio of the two numbers (in arbitrary order) and use the result as one entry in the null distribution. Then return back to the first step and repeat.

We repeated the above scheme 10^5 times to obtain a p-value associated with the ratio R.

Supplementary References

1. Boďová, K, Mitchell, G. J., Harpaz, R., Schneidman, E. & Tkačik, G. Probabilistic models of individual and collective animal behavior. *PLoS ONE* **13**(3): e0193049 (2018).

2. Gillespie, D. T. Exact stochastic simulation of coupled chemical reactions. *J. Phys. Chem.* **81**, 2340-2361 (1977).

3. Kurze, C., Jenkins, N. E. & Hughes, D. P. Evaluation of direct and indirect transmission of fungal spores in ants. *J. Invertebr. Pathol.* **172**, 107351 (2020).

4. York, D. Least-squares fitting of a straight line. *Can. J. Phys.* **44**, 1079-1086 (1966).
