# Supplementary material for: Dynamic pathogen detection and social feedback shape collective hygiene in ants
Source: Nat Commun. 2023 Jun 3;14:3232. doi: 10.1038/s41467-023-38947-y (PMC10239465; doi:10.1038/s41467-023-38947-y)
Supplement: Supplementary file 3 — Reporting Summary [file 41467_2023_38947_MOESM3_ESM.pdf]

nature portfolio

Corresponding author(s): Gasper Tkacik, Sylvia Cremer

Last updated by author(s): May 13, 2023

# Reporting Summary

Nature Portfolio wishes to improve the reproducibility of the work that we publish. This form provides structure for consistency and transparency in reporting. For further information on Nature Portfolio policies, see our Editorial Policies and the Editorial Policy Checklist.

## Statistics

For all statistical analyses, confirm that the following items are present in the figure legend, table legend, main text, or Methods section.

| n/a | Confirmed | |
|---|---|---|
| ☐ | ☒ | The exact sample size (*n*) for each experimental group/condition, given as a discrete number and unit of measurement |
| ☐ | ☒ | A statement on whether measurements were taken from distinct samples or whether the same sample was measured repeatedly |
| ☐ | ☒ | The statistical test(s) used AND whether they are one- or two-sided<br>*Only common tests should be described solely by name; describe more complex techniques in the Methods section.* |
| ☐ | ☒ | A description of all covariates tested |
| ☐ | ☒ | A description of any assumptions or corrections, such as tests of normality and adjustment for multiple comparisons |
| ☐ | ☒ | A full description of the statistical parameters including central tendency (e.g. means) or other basic estimates (e.g. regression coefficient) AND variation (e.g. standard deviation) or associated estimates of uncertainty (e.g. confidence intervals) |
| ☐ | ☒ | For null hypothesis testing, the test statistic (e.g. *F*, *t*, *r*) with confidence intervals, effect sizes, degrees of freedom and *P* value noted<br>*Give P values as exact values whenever suitable.* |
| ☒ | ☐ | For Bayesian analysis, information on the choice of priors and Markov chain Monte Carlo settings |
| ☐ | ☒ | For hierarchical and complex designs, identification of the appropriate level for tests and full reporting of outcomes |
| ☐ | ☒ | Estimates of effect sizes (e.g. Cohen's *d*, Pearson's *r*), indicating how they were calculated |

*Our web collection on statistics for biologists contains articles on many of the points above.*

## Software and code

Policy information about availability of computer code

| | |
|---|---|
| Data collection | Primer3Plus software was used for design of primers and probes, ddPCR data analysis was performed by QuantaSoft™ Analysis Pro Software (Bio-Rad, version 1.0), Streampix 5 digital video recording software (NorPix, Inc.) was used for acquisition of video data and Solomon Coder (version beta 17.0) for behavioral annotation from the videos, and Leica Application Suite Advanced Fluorescence 2.3.0 for fluorescence microscopy. |
| Data analysis | Statistical data analyses were performed in R v.4.2.1 (packages: stats (base package of R v.4.2.1, lme4 1.1-29, multcomp 1.4-19, DHARMa 0.4.5, glmmTMB 1.1.3, effectsize 0.7.0, rcompanion 2.4.15, rstatix 0.7.0, tidyverse 1.3.1, forcats 0.5.1, data.table 1.14.2, stringer 1.4.0, ggplot2 3.3.6, cowplot 1.1.1, ggpubr 0.4.0, scales 1.0.0) and Matlab v.2016b (MathWorks). |

For manuscripts utilizing custom algorithms or software that are central to the research but not yet described in published literature, software must be made available to editors and reviewers. We strongly encourage code deposition in a community repository (e.g. GitHub). See the Nature Portfolio guidelines for submitting code & software for further information.

## Data

Policy information about availability of data

All manuscripts must include a data availability statement. This statement should provide the following information, where applicable:

- Accession codes, unique identifiers, or web links for publicly available datasets
- A description of any restrictions on data availability
- For clinical datasets or third party data, please ensure that the statement adheres to our policy

Source data are provided with this manuscript.

## Human research participants

Policy information about studies involving human research participants and Sex and Gender in Research.

| | |
|---|---|
| Reporting on sex and gender | N/A |
| Population characteristics | N/A |
| Recruitment | N/A |
| Ethics oversight | N/A |

Note that full information on the approval of the study protocol must also be provided in the manuscript.

# Field-specific reporting

Please select the one below that is the best fit for your research. If you are not sure, read the appropriate sections before making your selection.

☐ Life sciences ☐ Behavioural & social sciences ☒ Ecological, evolutionary & environmental sciences

For a reference copy of the document with all sections, see nature.com/documents/nr-reporting-summary-flat.pdf

# Ecological, evolutionary & environmental sciences study design

All studies must disclose on these points even when the disclosure is negative.

Study description

We observed the behaviour of ants in groups of six individually colour-coded workers, of which four remained untreated and two were treated with either a high or a low dose of pathogenic fungal spores, or a sham control. The all-pairwise combinations of these three individual ant treatments resulted in six different group treatments (FF, Ff, ff, FC, fC, CC). We filmed each group 30 min before and 90 min after treatment. At the end of the experiment, we quantified spore load separately for each ant's head and body (by ddPCR), distinguishing the origin of the spores from the two treated ants by differential fluorescent labeling (eGFP, mRFP1), as well as the spores expelled by the ants as disinfected pellets. We determined the behavioural changes of the ants' individual and social hygiene behaviours due to treatment (Wilcoxon paired test pre vs post treatment). For the nestmates, we test whether their selfgrooming time depended on the treatment of the group member they previously performed grooming to, or received grooming from (Kruskal-Wallis tests). We further inferred the spore load of each ant over the course of the experiment using the Michaelis Menten kinetics. For each grooming event of the untreated nestmates in groups where they could choose between two spore-treated ants, we determined if they preferentially targeted the ant with the higher or lower current spore load (bootstrapped Kolmogorov-Smirnov test), and whether the duration of grooming depended on the relative distribution of pathogen load between the two ants (pairwise Kruskal-Wallis tests). We developed multiple stochastic transition models of the ant behavior and formulated the full stochastic behavioral model for the group of ants. We tested, which model could best predict our experimental data using model selection by (a) minimizing log-likelihood of the transition model on the experimental dataset, (b) minimizing prediction error between full stochastic simulations and experimental data for 5 independent replicate simulation sets for the time-resolved activity across all ant classes (F,f,C,N) and treatment combinations. The model selection identified best model components including the decision variables and the best performing grooming rules. We also observed two groups of ants to exemplify the sequences of grooming and non-grooming interactions (antennations) performed by each ant. We determine how the nestmates' grooming preference depends on the spore-load difference between the two treated ants, and assess the time-dependent effect of received grooming on own grooming performance for the differently-treated ants (both by Spearman-Rank correlation). We further model how spore removal efficiency in the group depends on group size and individual decision rules (stochastic simulation). We determine the nestmates' grooming preference and how well it correlates with spore load removal of the group (linear functional model using estimated detection error; 5 simulations per replicate). We then performed an experiment, in which the ants were prevented from performing grooming choice and compare spore removal between groups with and without choice (one-sided test based on the bootstrapped null distribution).

Research sample

For our study, we used the common fungal entomopathogen Metarhizium and its natural host of Lasius ants, which is a well-established model system for the study of social immunity in ants (see e.g. Cremer et al. 2018 Annu Rev Entomol). As pathogen, we used Metarhizium robertsii, strain ARSEF 2575 with either an integrated green (eGFP) or red (mRFP1) fluorescent label (obtained from M. Bidochka, Brock University), to be able to simultaneously determine the transmission of the pathogen from two pathogen-

| | |
|---|---|
| | exposed individuals through the group of ants, in dependence of the observed ant behaviour. As host, we used the invasive garden ant, Lasius neglectus. As a representative for its introduced supercolonial populations, we collected the ants in the Botanical Garden in Jena, Germany (N 50° 55.910 E 11° 35.140) in June 2015 and in September 2022. The field-collected insects were brought back to the laboratory for rearing in a large stock colony, from which we used workers in 2016 for the experiment. The choice removal experiment was performed in 2022 with the ants collected the same year. |
| Sampling strategy | We observed ants interacting in groups of six, which is a large enough group size to allow for collective effects to emerge (Ulrich et al, 2018, Nature) and small enough to manually score all self-directed and social behaviours of each individually colour-coded ant in frame-based temporal resolution (videos taken at 15 frames per second) over the whole experimental period of 2 hours (n=99 groups, 594 ants). Each group was filmed for 30 min before treatment to obtain the untreated baseline behavior of each ant. As previous work revealed that L. neglectus ants exhibit hygiene behaviors already within the first hours after pathogen contact (e.g. Konrad et al, 2012 PLoSBiol), we observed behaviors immediately after reintroduction of our two treated ants to the group. The experiments were filmed for 90 min and the behavioural changes in reaction to treatment (by all ants in the group, also the untreated nestmates) were scored. Our three individual ant treatments (high or low fungal load, sham control) led to six group treatments by all-pairwise combinations of the of the two treated ants per group, which allowed us to study the ants' reaction to different overall group pathogen load and load differences between the two treated individuals. Each treatment was represented by 16-17 biological replicates, so that we achieved individual observations of a total of 594 workers, of which 66 were treated with high F, 65 with low f fungal load, 67 with a control C, and 396 remained untreated nestmates N; Suppl. Table 1). To avoid pseudoreplication in our behavioural observations, we obtained a single value per individual treatment and replicate, by averaging the values of all ants in the same group which had received the same individual treatment (i.e. one average value for the two F-individuals in FF, the two f-individuals in ff, the two C-individuals in CC, and the four N in any group). As we observed multiple behaviours (selfgrooming head and body, poison uptake, grooming performed and received) by each individual, we corrected our statistical inferences for multiple testing. In total, we observed 8129 grooming decisions by the 328 nestmate ants in the 82 replicates with at least one spore-treated individual, which we used to derive individual rules of the ants' grooming decisions in relation to pathogen load and social interactions. We provide two-sided, exact p-values (unless p<1e-11), after correction for multiple testing following the Benjamini-Hochberg correction (to protect against a FSD of 5%), where appropriate. We performed a follow-up experiment to test for our a priori hypothesis derived from the main experiment that grooming choice has a functional benefit in allowing for higher spore removal. We here compare the spore removal of the groups with choice (4 nestmates with 2 treated individuals, in 4 situations of differing pathogen load differences between the two treated ants, based on three spore concentrations and a sham treatment) and that of groups, in which choice was prevented (2 nestmates with 1 treated individual, in 3 situations with the three spore concentrations). We analysed each situation in 14 replicates, ie. a total of 98 groups and 462 ants. We performed a one-sided test on the ratio of the removed spores in the groups with choice to the groups without choice combined for the different initial spore load differences, using bootstrapping. |
| Data collection | The experiments were performed and behaviours annotated by BC and AVG. Primers were designed and PCRs performed by AVG. |
| Timing and spatial scale | The ants for the main experiment were collected from the Botanical Garden in Jena, Germany, in 2015 and reared in the laboratory before running the experiment in a single block over 6 experimental days in 2016. In 2016, 1265 multiplex ddPCR samples (594 head, 594 body samples and 77 pellet pools) were run (each for both fungal spore labels). In 2017, the 198 hours of video were manually scored with frame-level precision (15 fps, summing up to > 10.5 Mio individual frames), each analyzed for all six individual ants per group. From 2018, data were analyzed statistically and modeling was performed. In 2022, the choice removal experiment was performed with ants collected that same year from the same population. |
| Data exclusions | From the original 108 replicates (18 per treatment group), 9 had to be excluded for technical errors occurring during experimental performance, so that we obtained 16 to 17 replicates for each of the six treatment groups (n = 99 replicate groups of six ants; Suppl. Table 1). For the choice removal experiment, we had originally obtained 15-16 replicates per situation, yet were restricted by our bootstrapping approach by the greatest common even number across situations, and thus only included replicates 1-14 per situation into the analysis. |
| Reproducibility | Both experiments were each run in a single block and reproducibility was obtained by a high simultaneous replication as detailed above. Simulations for the model selection were based on 5 replicates each, leading to a robust modeling dataset. |
| Randomization | Groups of six ants were randomly assigned to one of six group treatments (FF, Ff, ff, FC, fC, CC), and each ant per group was differentially color-coded. Individual treatment (F, f, C) was applied to the ants, such that color could not be used to identify ant treatment across groups. In groups where both treated ants received spores, the distinct spore labels (mRFP1 and eGFP), were assigned in a randomised and balanced manner, so that both labels were represented equally within and across individual and group treatments (detailed in Suppl. Table 1). Replicates and treatments were evenly distributed between observers for scoring. In the choice removal experiment, ants were equally assigned randomly to either a situation with or without choice, and individual treatment. |
| Blinding | Ant treatment was applied independent of ant color-code, so that no association could be made by the observer between ant color and treatment, neither within nor across replicates and treatment groups. Ant color-to-treatment assignment was also not revealed in the video, ensuring bias-free behavioral scoring. Similarly, samples for DNA extraction and further spore quantification by PCR did not contain information of the ant's treatment. |

Did the study involve field work? ☐ Yes ☒ No

# Reporting for specific materials, systems and methods

We require information from authors about some types of materials, experimental systems and methods used in many studies. Here, indicate whether each material, system or method listed is relevant to your study. If you are not sure if a list item applies to your research, read the appropriate section before selecting a response.

## Materials & experimental systems

| n/a | Involved in the study |
| --- | --- |
| ☒ | ☐ Antibodies |
| ☒ | ☐ Eukaryotic cell lines |
| ☒ | ☐ Palaeontology and archaeology |
| ☐ | ☒ Animals and other organisms |
| ☒ | ☐ Clinical data |
| ☒ | ☐ Dual use research of concern |

## Methods

| n/a | Involved in the study |
| --- | --- |
| ☒ | ☐ ChIP-seq |
| ☒ | ☐ Flow cytometry |
| ☒ | ☐ MRI-based neuroimaging |

# Animals and other research organisms

Policy information about studies involving animals; ARRIVE guidelines recommended for reporting animal research, and Sex and Gender in Research

| | |
| --- | --- |
| Laboratory animals | The insects (Lasius neglectus ants) used in our experiments stem from a field-collected supercolony (Jena, Germany), from where we collected queens, workers and brood to rear them in the laboratory under a temperature and light cycle reflecting natural seasons, until the experiments, which were performed 3 to 13 months after collection (July 2016 for the collection in June 2015 and December 2022 for the collection in September 2022). Workers are produced in spring and over the summer and are known to overwinter. For the experiments, we sampled workers from chambers inside the nest to avoid old foragers. We also did not sample freshly emerged individuals (callows, which can be distinguished by their pale colour due to missing melanisation and sclerotisation of the cuticle). |
| Wild animals | We used workers of the invasive garden ant, Lasius neglectus, in our experiments. We collected the ants from their nests in the soil in their introduced supercolonial population in the Botanical Garden in Jena, Germany (N 50° 55.910 E 11° 35.140), which has been detected in 1997 (Ugelvig et al. 2008 BMC Biol). Due to the intra-nest mating of newly produced daughter queens (once per year, in the summer) and their coexistence with already older reproductive queens, supercolonies are quasi-immortal. We collected queens, workers and brood in both June 2015 and September 2022, transported them back to the laboratory in plastic boxes, and reared them in the laboratory until their use in the experiments (performed 13 months after field collection for the main experiment in July 2016, and 3 months after collection for the choice removal experiment in December 2022). Ants were frozen at the end of the experiment, for molecular analysis of their pathogen load. |
| Reporting on sex | As ants are Hymenoptera, all worker ants are female. |
| Field-collected samples | After collection, ants were reared until use in the experiments in an incubator reflecting the yearly temperature and a circadian day/night light cycle. For the experiments, the workers were taken from their stock colony, placed in petri dishes with humidified plastered floor and observed in a temperature- and humidity-controlled room at 23 °C and 65% RH. |
| Ethics oversight | We used ants (insects, invertebrates) as our study animals. The study was performed on workers of an invasive ant species, the invasive garden ant, Lasius neglectus. Collection of this unprotected species from the field in Germany in 2015 and 2022 was in compliance with international regulations, such as the Convention on Biological Diversity and the Nagoya Protocol on Access and Benefit-Sharing (ABS), yet not requiring specific permits. Transport to and rearing of the ants in the laboratory, as well as all experimental work followed European and Austrian law and institutional ethical guidelines of the Institute of Science and Technology Austria (ISTA). |

Note that full information on the approval of the study protocol must also be provided in the manuscript.
